# Oral administration of turmeric-derived exosome-like nanovesicles with anti-inflammatory and pro-resolving bioactions for murine colitis therapy

**DOI:** 10.1186/s12951-022-01421-w

**Published:** 2022-04-29

**Authors:** Cui Liu, Xiangji Yan, Yujie Zhang, Mei Yang, Yana Ma, Yuanyuan Zhang, Qiuran Xu, Kangsheng Tu, Mingzhen Zhang

**Affiliations:** 1 School of Basic Medical Sciences, Xi’an Key Laboratory of Immune Related Diseases, Xi’an Jiaotong University, Xi’an, 710061 Shaanxi China; 2 Key Laboratory of Environment and Genes Related to Diseases, Ministry of Education, Xi’an Jiaotong University, Xi’an, 710061 Shaanxi China; 3 Laboratory of Tumor Molecular Diagnosis and Individualized Medicine of Zhejiang Province, Zhejiang Provincial People’s Hospital, Affiliated People’s Hospital, Hangzhou Medical College, Hangzhou, 310014 Zhejiang China; 4 Department of Hepatobiliary Surgery, The First Affiliated Hospital of Xi’an Jiaotong University, Xi’an, 710061 Shaanxi China; 5 Institute for Biomedical Sciences, Center for Diagnostics and Therapeutics, Digestive Disease Research Group, Georgia State University, Atlanta, GA 30302 USA

**Keywords:** Turmeric-derived nanoparticles, Ulcerative colitis, Nanotherapeutics, Oral administration, NF-κB pathway

## Abstract

**Background:**

Ulcerative colitis (UC) is an inflammatory bowel disease (IBD) characterized by diffuse inflammation of the colonic mucosa and a relapsing and remitting course. The current therapeutics are only modestly effective and carry risks for unacceptable adverse events, and thus more effective approaches to treat UC is clinically needed.

**Results:**

For this purpose, turmeric-derived nanoparticles with a specific population (TDNPs 2) were characterized, and their targeting ability and therapeutic effects against colitis were investigated systematically. The hydrodynamic size of TDNPs 2 was around 178 nm, and the zeta potential was negative (− 21.7 mV). Mass spectrometry identified TDNPs 2 containing high levels of lipids and proteins. Notably, curcumin, the bioactive constituent of turmeric, was evidenced in TDNPs 2. In lipopolysaccharide (LPS)-induced acute inflammation, TDNPs 2 showed excellent anti-inflammatory and antioxidant properties. In mice colitis models, we demonstrated that orally administrated of TDNPs 2 could ameliorate mice colitis and accelerate colitis resolution via regulating the expression of the pro-inflammatory cytokines, including TNF-α, IL-6, and IL-1β, and antioxidant gene, HO-1. Results obtained from transgenic mice with NF-κB-RE-Luc indicated that TDNPs 2-mediated inactivation of the NF-κB pathway might partially contribute to the protective effect of these particles against colitis.

**Conclusion:**

Our results suggest that TDNPs 2 from edible turmeric represent a novel, natural colon-targeting therapeutics that may prevent colitis and promote wound repair in colitis while outperforming artificial nanoparticles in terms of low toxicity and ease of large-scale production.

**Supplementary Information:**

The online version contains supplementary material available at 10.1186/s12951-022-01421-w.

## Background

Inflammatory bowel disease (IBD), mainly including ulcerative colitis (UC) and Crohn’s disease (CD), is a chronic, nonspecific intestinal inflammatory disease that affects a growing number of people worldwide. IBD has the characteristics of diarrhea, pain, ROS generation, oxidative stress imbalance, and elevated colon cancer risk [1, 2]. UC is characterized by a persistent disease course with relapse and remission. Currently, the primary goal for UC treatment is to maintain the long-term remission of inflammation [3, 4]. The etiology and pathogenesis of UC are still unclear. Modern medicine believes that it is under the combined influence of genetics, environment, psychology, and other factors, resulting in neuroendocrine dysfunction, intestinal mucosal barrier damage, and immune imbalance, thereby causing intestinal mucosal damage locally [5–7]. The current medicines commonly used to treat UC are aminosalicylates, corticosteroids, and immunosuppressants. Under some specific conditions, biologics are alternative [8–10]. Nevertheless, affected by uncontrollable factors, the current therapeutic approaches cannot control UC thoroughly [11, 12]. Many UC patients have gastrointestinal obstruction, perforation, bleeding, abscesses, fistulas, and even colon tumors. Therefore, we urgently need to develop better therapeutics for UC, especially those that can act locally in the inflamed colon, without whole-body exposure and the associated side effects.

Recently, drug delivery systems based on novel nanoparticles with colon targeting capacity have attracted substantial attention for UC treatment [13–16]. These delivery systems offer several advantages, including (1) increased drug concentration at the inflamed colon, which can maximize drug efficacy and prolong pharmacological activity; and (2) targeted delivery, which can wholly or partially prevent the drug from degrading and losing effectiveness before it reaches the active site, and potentially reduce the dosing frequency and/or minimize systemic side effects [17, 18]. Nanoparticle carriers may also be used to modify some properties of the drug, including its solubility, stability, and immunogenicity [19, 20]. However, those artificial nanoparticles encounter several tricky issues for their clinical applications, including (1) the potential in vivo toxicities, (2) the high cost for mass production, and (3) potential adverse environmental impacts [21–24]. In contrast, plant-derived edible nanoparticles with unique properties have good application prospects for colitis-targeted therapy [25–28].

Turmeric, perennial plant (*Curcuma longa*) of the ginger family, is traditional Chinese medicine, which has been used for conditions involving hypolipidemic, antineoplastic, anti-inflammatory, and effects on the cardiovascular system, etc. [29–33]. Curcumin is a natural hydrophobic polyphenol from turmeric to exert its pharmacological effects. It has pharmacological effects such as antioxidant, anti-inflammatory, anti-angiogenesis, anti-tumor, and has no noticeable adverse reactions [34–36]. Additionally, curcumin is edible, inexpensive, and available in bulk. Curcumin has been paid great attention to these incomparable properties, and numerous therapeutic applications against multiple diseases have been developed [37, 38]. Inspired by the medicinal values of turmeric and our previous research [39–42], we hypothesized that turmeric-derived nanoparticles have great potential to be applied for colitis therapy.

**Fig. 1:**
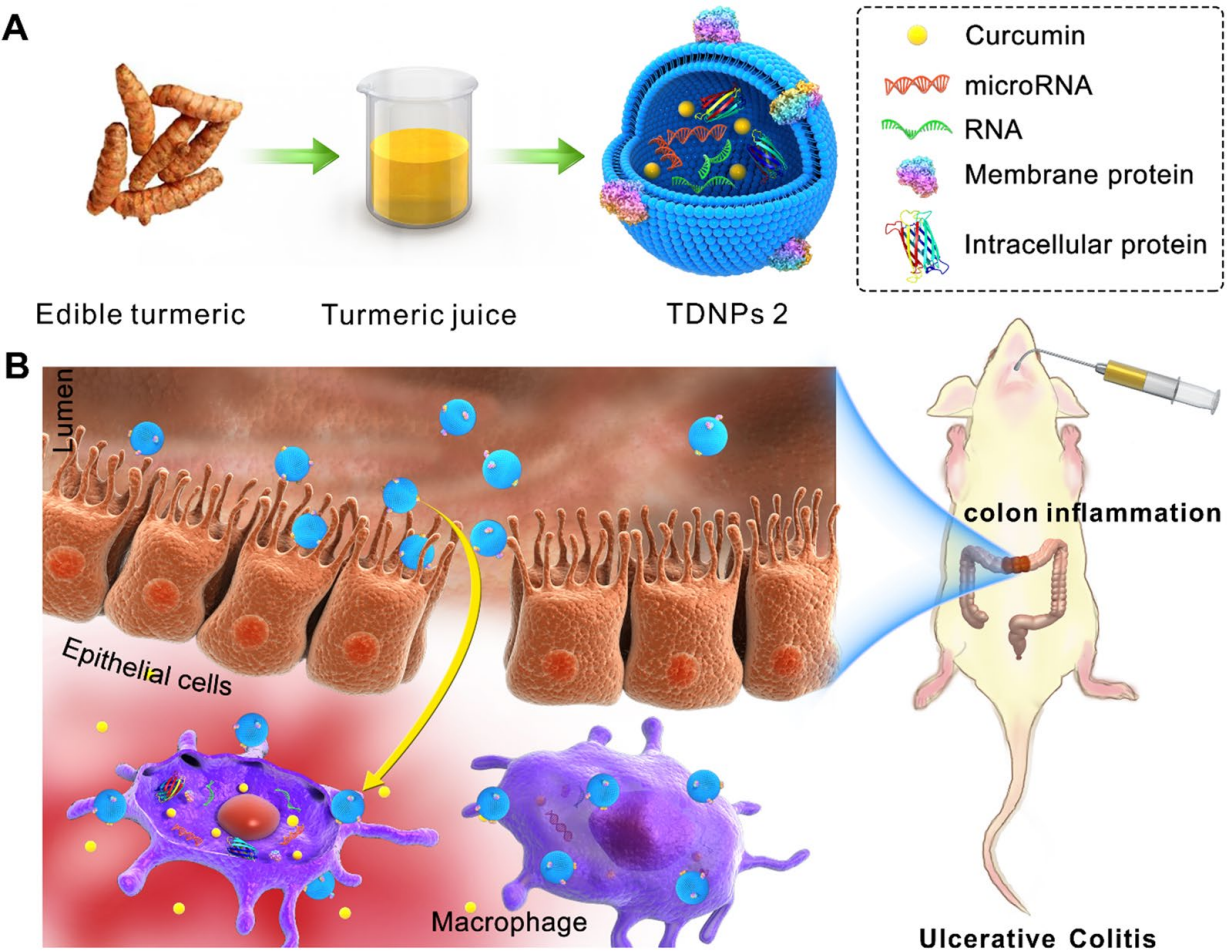
Schematic illustration of turmeric-derived nanoparticles (TDNPs 2) isolation and targeted ulcerative colitis (UC) therapy via oral administration. **A** TDNPs 2 were isolated and purified from edible turmeric by ultracentrifugation and sucrose gradient centrifugation. **B** By oral administration, TDNPs 2 accumulated at inflamed colon and exerted UC therapeutic effect

In this study, a specific population of turmeric-derived nanoparticles (TDNPs 2) were isolated and purified by ultracentrifugation and sucrose gradient centrifugation. We investigated their site-specific targeting to inflamed colon and therapeutic effects against colitis following oral administration (Fig. 1). TDNPs 2 has a hydrodynamic size of 178 nm with negative zeta potential (− 21.7 mV), with mass spectrometry and HPLC analysis, specific lipids, proteins, and bioactive constituent-curcumin were identified in TDNPs 2. In LPS-induced acute inflammation, TDNPs 2 showed excellent anti-inflammatory and antioxidant potentials. In mice models of colitis, orally administration of TDNPs 2 preferentially localized to the inflamed colon and were mainly internalized by colonic epithelial cells and macrophages. TDNPs 2 alleviated mice colitis induced by DSS and promoted inflammation resolution of the inflamed colon by attenuating damaging factors while promoting protective factors. TDNPs 2 inactivated the NF-κB pathway, potentially explaining their protective effect against colitis. Our results collectively show that TDNPs 2 represent a novel therapeutic approach for colitis with inflammation prevention and resolution functions.

## Materials and methods

### Cell culture

Colon-26 cells used in the experiment were cultured by RPMI 1640 basal medium, Caco-2BBE, and RAW 264.7 cells in our study were cultured by basal DMEM medium with high glucose. Heat-inactivated fetal bovine serum (10%) and penicillin/streptomycin (100 U/ml, 100 U/ml, respectively) were added in the medium to prepare the complete medium. All the cells were cultured in dishes or flasks according to the pre-set experiments at 37 °C in a humidified atmosphere containing 5% CO_2_.

### Animals

The FVB/NJ female mice and the NFκB-RE-Luc transgenic female mice with 6–8 weeks were provided by Jackson Laboratories (Bar Harbor, ME, USA) and Taconic Biosciences (New York, NY, USA), respectively. When the mice were received, they were housed under specific pathogen-free (SPF) conditions to adapt to the environment. All animal procedures were performed following the Institutional Animal Care and Use Committee (IACUC) guidelines of Georgia State University and Xi'an Jiaotong University.

### Mice colitis and colitis wound-healing models

To establish the mice colitis model, FVB/NJ mice were given 3% (w/v) dextran sulfate sodium (DSS) in their drink water for continuous 7 days. Mice were orally administrated with TDNPs 1 or TDNPs 2 (3 mg/dose) each day for successive 7 days. During the experiment, mice feces in each group were collected, and Lcn-2 in feces was quantified to monitor the development and remission of inflammation. On day 7, mice were injected with a XenoLight RediJect inflammation probe for chemiluminescence imaging by IVIS imaging system. Finally, mice were sacrificed, and colon tissues were obtained for the following experiments, such as RNA and histopathological analyses.

To generate colitis wound-healing model, FVB/NJ mice were given 3% (w/v) dextran sulfate sodium (DSS) in their drink water for continuous 7 days to induce colitis. On day 7, mice were orally treated with TDNPs 2 (3 mg/dose) each day or with regular water without any treatment (control group) for 1 week. As in the colitis mice model, feces were collected to quantify Lcn-2, spleen weight was captured and compared, mice inflammation was monitored in vivo by XenoLight RediJect inflammation probe via chemiluminescence imaging, colons were captured for MPO and RNA analysis.

### Statistical analyses

Analyses of variance (ANOVA) and t-tests analysis between groups were carried out by GraphPad Prism 7 software to determine statistical significance (*p < 0.05, **p < 0.01, ***p < 0.001).

## Results

### Characterization of turmeric-derived nanoparticles (TDNPs)

**Fig. 2:**
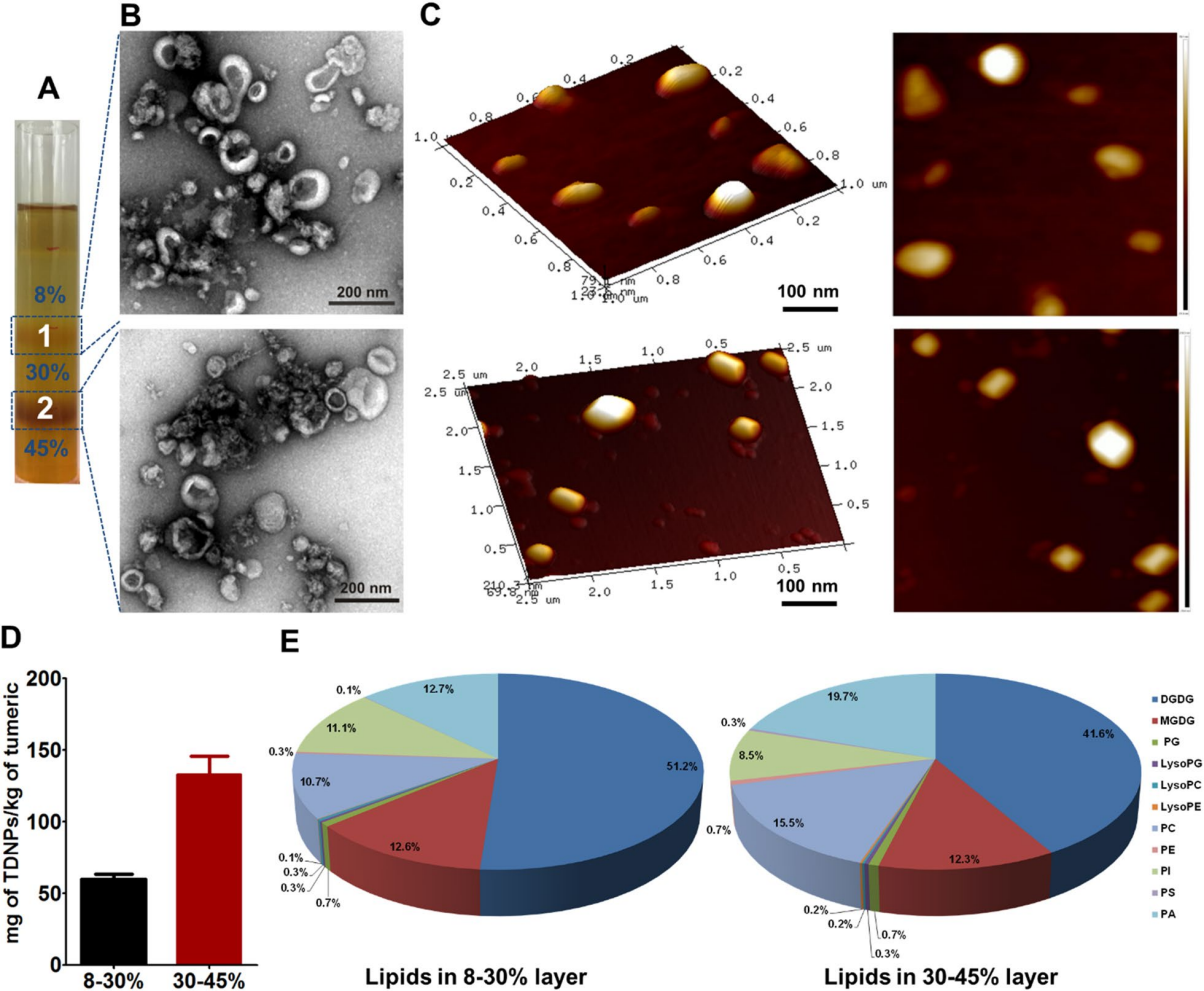
Characterization of TDNPs. **A** TDNPs were isolated and purified by sucrose gradient ultracentrifugation, band 1 from 8%/30% interface was named TDNPs 1, and band 2 from 30%/45% interface was named TDNPs 2. **B** Transmission Electron Microscopy (TEM) to characterize the morphology and size. **C** TDNPs were also identified by Atomic Force Microscopy (AFM). **D** Yields of TDNPs 1 and TDNPs 2 from turmeric were calculated and compared (n = 5). **E** Putative lipid species between TDNPs 1 and TDNPs 2 were compared

To isolate TNDPs, plant turmeric was homogenized by an extractor followed by a sucrose gradient ultracentrifugation method established in our previous work with slight modification [43, 44]. Band 1 from the sucrose gradient interfaces of 8/30% was named TNDPs 1, and band 2 from the sucrose gradient interfaces of 30/45% was named TNDPs 2 (Fig. 2A). The dynamic light scattering (DLS) technique was used to determine the size and zeta potential of TNDPs. Results showed that the hydrophobic sizes of TNDPs 1 and TNDPs 2 were 204.6 nm and 177.9 nm, respectively. The zeta potential was about − 21.3 mV and 21.7 mV for them (Additional file 1: Fig. S1). To observe the ultrastructure and morphology, TEM and AFM were employed. TEM results showed that both TDNPs 1 and TDNPs 2 possessed the structure of saucer-shaped or hemispherical with a concave side, which was similar to the structure of exosomes from mammalian cells (Fig. 2B). AFM can characterize the sample morphology with high resolution and obtain the physical properties such as sample viscosity, softness, and hardness. The AFM results indicated a non-homogenous surface, which should attribute to the contents such as proteins and mRNA enclosed inside the highly-dense lipid membrane (Fig. 2C). About 26.45 ± 10.51 mg of GDNPs 1 and 132.87 ± 22.24 mg of GDNPs 2 were obtained from a starting material of 1000 g turmeric with a considerable yield (Fig. 2D). Collectively, two nanoscaled TDNPs from turmeric were isolated successfully using the characterization methods for exosomes. Exosomes from mammalian cells are membrane-covered structures with a density in sucrose of 1.13–1.19 g/ml [45, 46]. According to this, TDNPs 2 from 30/45% sucrose gradient interface were referred to as turmeric-derived exosomes-like nanoparticles. Lipidomic results indicated that both TDNPs 1 and TDNPs 2 were enriched with digalactosyl diacylglycerol (DGDG) (51.2% and 41.6%, respectively), monogalactosyl diacylglycerol (MGDG) (12.6% and 12.3%, respectively), phosphatidylcholine (PC) (10.7% and 15.5%, respectively), phosphatidylinositol (PI) (11.1% and 8.5%, respectively) and phosphatidic acid (PA) (12.7% and 19.7%, respectively) (Fig. 2E, Additional file 1: Table S1). Proteomics analysis for TDNPs 1 and TDNPs 2 was also performed by LC–MS (Additional file 1: Table S2). In TDNPs 1, no protein was identified. In contrast, many proteins were found in TDNPs 2, such as cytosolic proteins and membrane proteins. Meanwhile, many uncharacterized proteins indicated the undiscovered function and relevance with TDNPs 2 or turmeric. It was revealed that turmeric possessed the function for chronic pain and inflammation because of its active ingredient curcumin, which had powerful biological properties such as targeting specific inflammatory cells and blocking certain enzymes that lead to inflammation. Using HPLC, we found that TDNPs 2 contained a higher level of curcumin (16.8 ± 3.4 µg/mg) than that in TDNPs 1 (8.6 ± 0.5 µg/mg), indicating TDNPs 2 might have their specific biochemical characteristics (Additional file 1: Fig. S2).

TDNPs should pass through the complex digestive tract and keep them stable for oral administration. We then evaluated their stability in a stomach-like solution (pH ~ 2.0) and a small-intestine-like solution (pH ~ 6.5), respectively (Additional file 1: Fig. S3). We found that the particle size of TDNPs 1 and TDNPs 2 was increased to 331.7 nm and 348.5 nm, respectively, under a stomach-like solution, and the size was further increased to 569.5 nm and 507.7 nm, respectively, under a small-intestine-like solution. Their zeta potential was changed from negative to weakly positive under a stomach-like solution, and this change was reversed to negative again under a small-intestine-like solution. Under different pH solutions, both TDNPs 1 and TDNPs 2 are still in nano-scale size, indicating their stability, and the zeta potential changes coincide with the natural properties of TDNPs.

### TDNPs 2 prevent LPS-induced macrophage inflammation

**Fig. 3:**
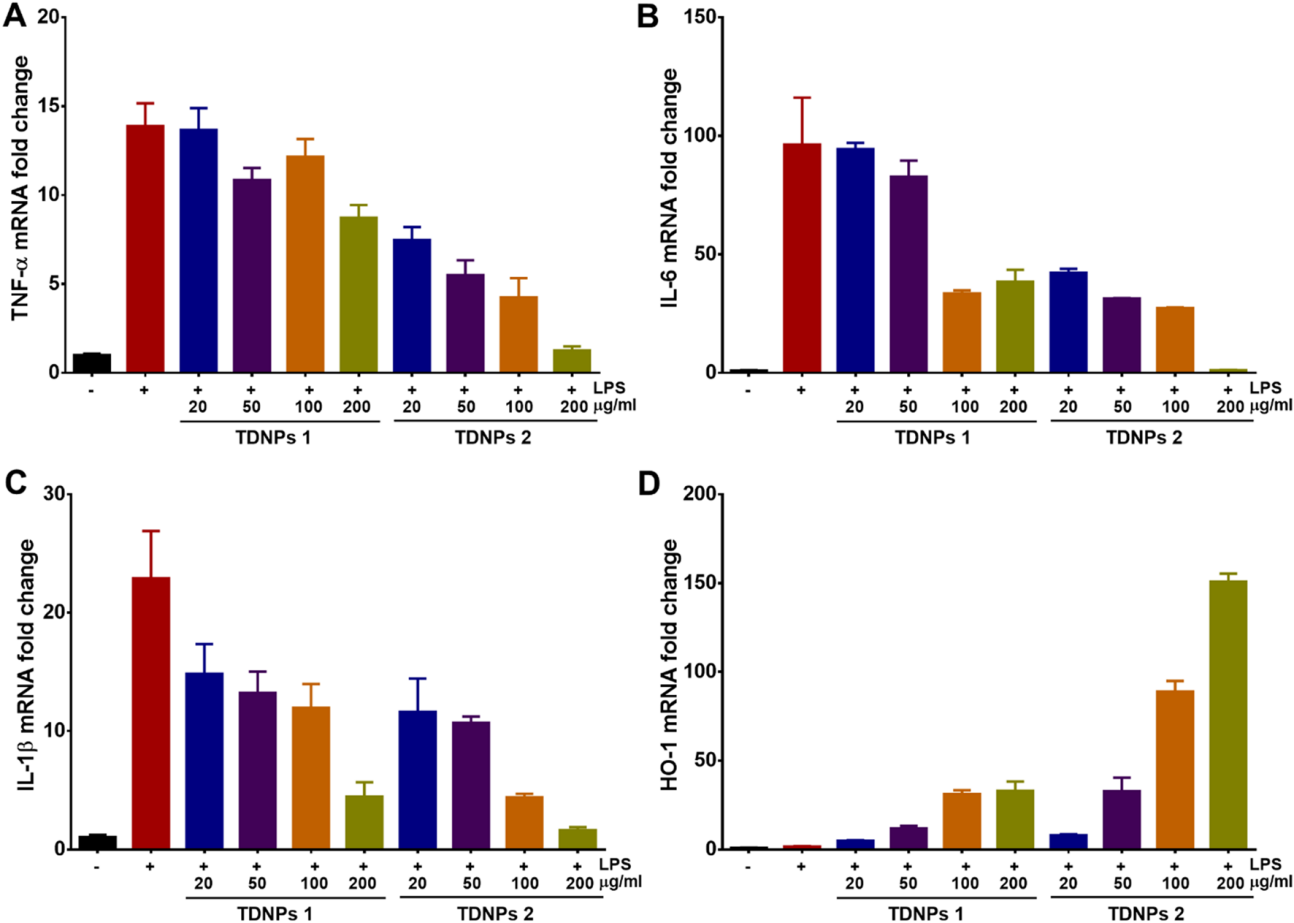
TDNPs prevent LPS-induced macrophage inflammation. **A–C** mRNAs expression of pro-inflammatory cytokines, TNF-α, IL-6 and IL-1β, respectively (n = 5). **D** mRNA expression of HO-1 (n = 5)

Curcumin is turmeric’s most important chemical component to exert pharmacological effects, such as antioxidant anti-inflammatory. TDNPs contained a high level of curcumin, and we speculated TDNPs would have similar anti-inflammatory and antioxidant functions. Lipopolysaccharide (LPS)-induced macrophage inflammation was used to investigate the effect of TDNPs in vitro. LPS can cause an acute inflammatory response by activating TLR4/MyD88 signaling pathway to produce many pro-inflammatory cytokines [47, 48]. TDNPs 1 and TDNPs 2 with different concentrations were incubated with activating RAW 264.7, the expressions of pro-inflammatory cytokines and HO-1 were evaluated by real-time PCR. Both TDNPs 1 and TDNPs 2 could decrease TNF-α, IL-6, and IL-1β expression and increase HO-1 expression to a certain degree, suggesting the presumptive anti-inflammatory and antioxidant potentials for TDNPs. Notably, TDNPs 2 displayed a super-duper concentration-dependent manner for regulating these genes expression (Fig. 3). These results should attribute to the high level of curcumin in TDNPs 2 and their natural properties. Taken together, TDNPs 2 have remarkable anti-inflammatory and antioxidant potentials than TDNPs 1. Coupled with the high yield from turmeric, TDNPs 2 are used in our followed experiments.

### TDNPs 2 preferentially localized to the inflamed colon

**Fig. 4:**
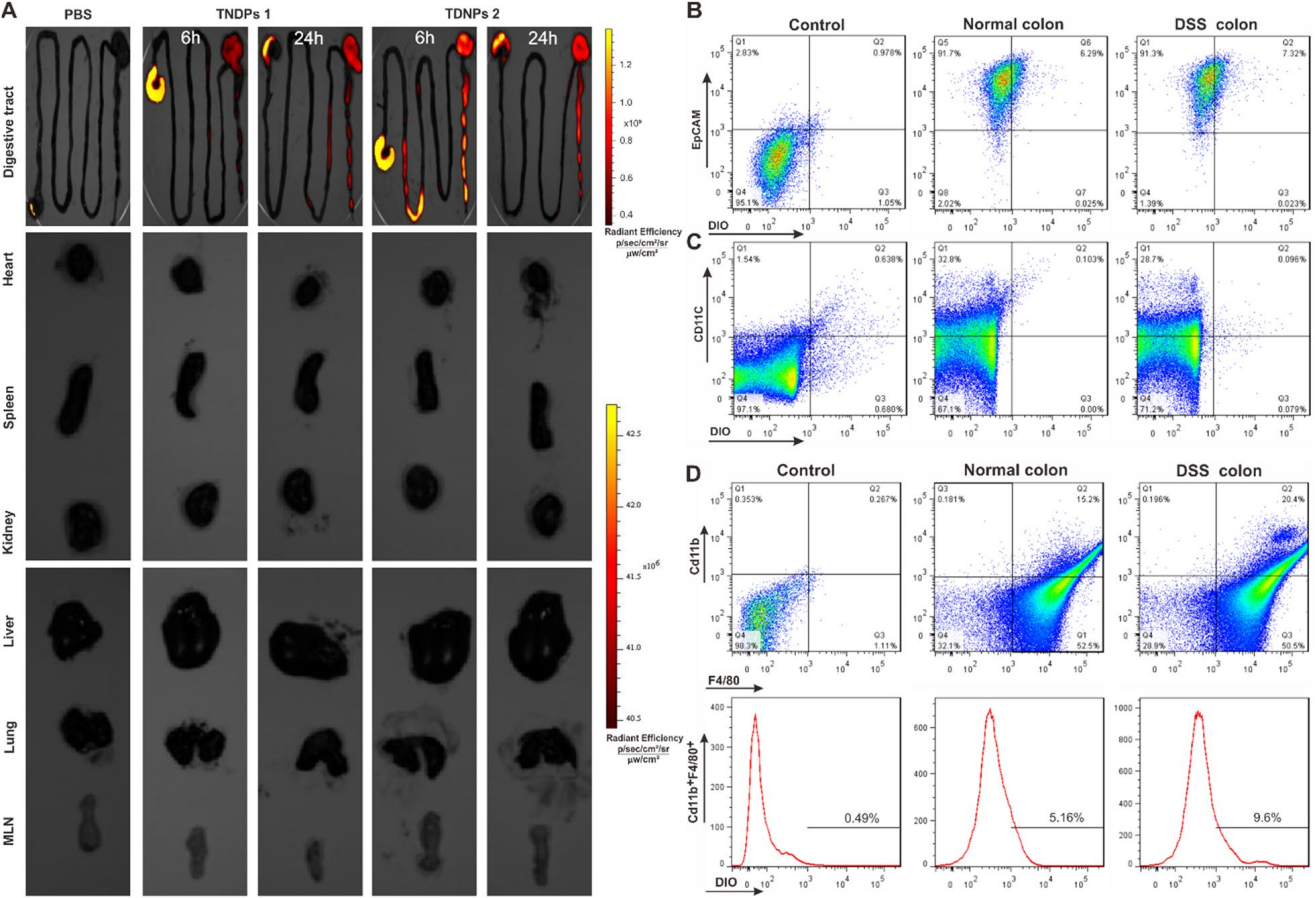
TDNPs 2 preferentially localized to the inflamed colon. **A** Digestive tract, mesenteric lymph nodes (MLN), and vital organs (Heart, liver, spleen, lung, kidney, and) were imaged by IVIS® Spectrum imaging system. **B–D** FACS was used to determine the population of cells to uptake DiO labeled TNDPs 2 (n = 3)

Oral administration is a convenient and economical delivery route, and it is widely used in disease treatment. Compared with other therapeutic routes, oral administration has many advantages, such as fewer adverse reactions, does not directly damage the skin or mucous membranes, economical and convenient for patients [49–51]. We next investigated the targeting ability of TDNPs 2 by oral administration. Colitis mice were gavaged with DiR-labeled TNDPs for 6 or 24 h. The digestive tract, mesenteric lymph nodes (MLN), and vital organs (heart, liver, spleen, lung, and kidney) were obtained for imaging by the IVIS® Spectrum imaging system (Fig. 4A). Results showed that DiR signal was mainly located in the digestive tract. It was delighted that there were strong DiR signals at the colon for TDNPs 2-treated mice compared with PBS and TDNPs 1-treated mice. In contrast, there was no conspicuous fluorescence of DiR in these vital organs and MLN. Collectively, TNDPs 2 are preferentially localized to the inflamed colon.

Next, different cells were isolated and sorted by flow cytometry to confirm which cell populations selectively uptake TDNPs 2. DiO-labeled TDNPs 2 were orally administrated to normal and colitis mice for 24 h, then cells from the colon were isolated for flow cytometry analysis. For colonic epithelial cells (EpCAM^+^), the population of DiO-positive cells in normal and colitis mice was 6.29% and 7.32%, respectively (Fig. 4B); For dendritic cells (CD11c^+^), this proportion was 0.103% and 0.096%, respectively (Fig. 4C); As for colonic macrophage cells (CD11b^+^F4/80^+^), this proportion in normal and colitis mice was 15.2% and 20.4%, respectively (Fig. 4D). All these results demonstrated that colonic epithelial cells (EpCAM^+^) and macrophage cells (CD11b^+^F4/80^+^) were the main cell populations to internalize TDNPs 2, and only a tiny percentage of dendritic cells (CD11c^+^) participated in the process. We also found that the rate of DiO-positive cells in colonic epithelial cells (EpCAM^+^) and macrophage cells (CD11b^+^F4/80^+^) was higher in colitis mice than in normal mice, indicating TDNPs 2 had an excellent targeting ability to colitis mice.

**Fig. 5:**
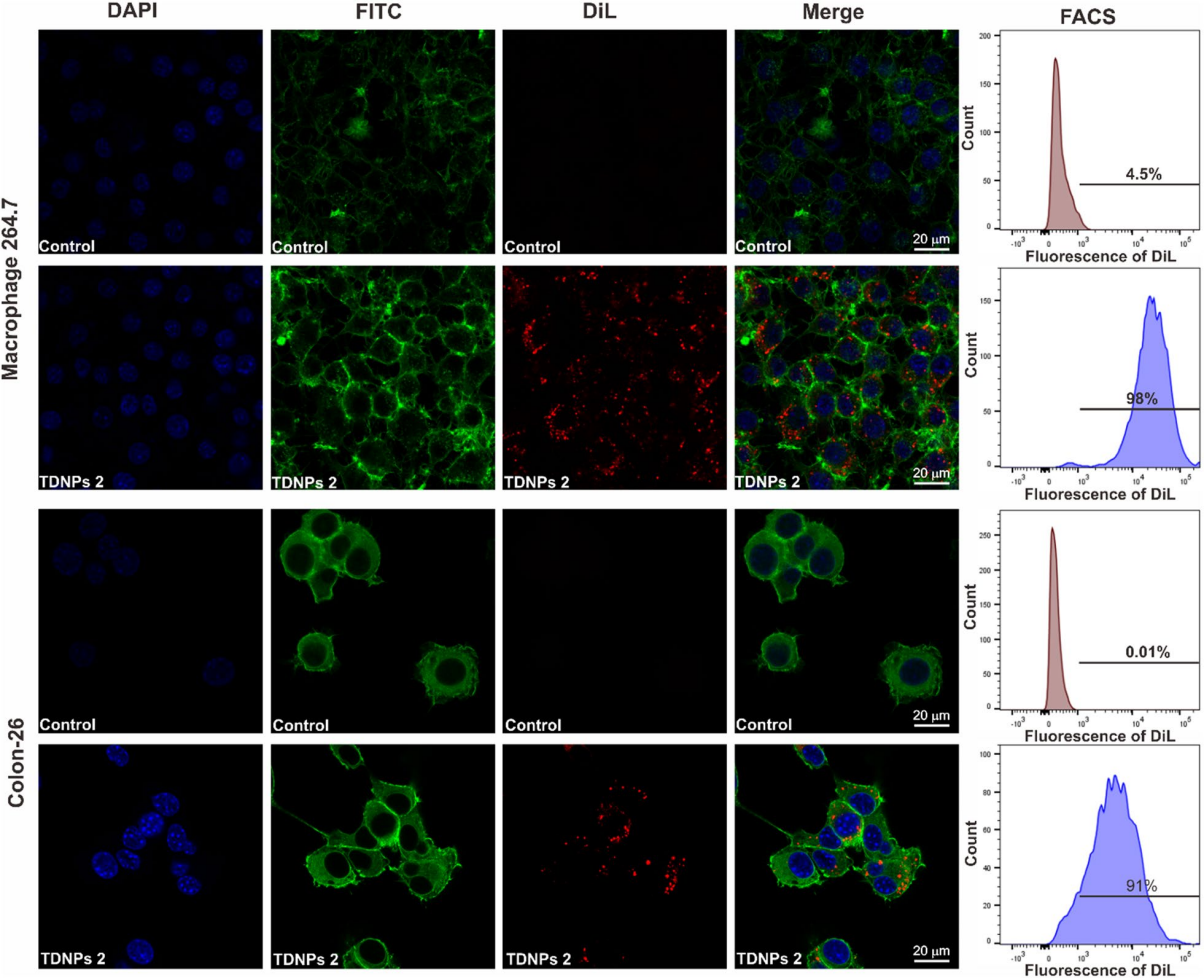
Cellular uptake of TNDPs 2 by macrophage 264.7 and colon-26 cells. The cell nucleus was stained by DAPI (Blue), the cytoskeleton was stained by FITC-phalloidin (Green), and TDNPs 2 were labeled by lipophilic carbocyanine dye, DiL (Red), scale bar: 20 μm

We incubated TNDPs 2 with RAW 264.7 and Colon-26 in vitro and investigated the cellular uptake of TNDPs 2 by these cells. TNDPs 2 were labeled by fluorescent dye-DiL with the emission peak at 565 nm. The confocal imaging showed lots of red dots (DiL labeled TNDPs 2) located in the cytoplasm for RAW 264.7 and Colon-26 cells. Flow cytometry analysis was consistent with confocal results, and the endocytosis efficiency for them was quantified up to 98% and 91%, respectively (Fig. 5). The internalization pathway(s) of TNDPs 2 in RAW 264.7 cells were also investigated by incubating with endocytosis inhibitors (Additional file 1: Fig. S4). Indomethacin could significantly inhibit the internalization of DiL-TNDPs 2, indicating caveolae-mediated endocytosis involved in TNDPs 2 cellular uptake. Other inhibitors, such as amiloride, cytochalasin D, and chlorpromazine, had a slight effect on the uptake of DiL-TDNPs 2 [52–55]. Effects of temperature on cellular uptake efficiency were also investigated. DiL-TDNPs 2 were incubated with RAW 264.7 cells under different temperatures (37, 20, and 4 °C), as shown in Additional file 1: Fig. S5, the uptake efficiency of DiL-TDNPs 2 by RAW 264.7 cells was significantly decreased under 4 and 20 °C when compared with the result under 37 °C condition, indicating that the uptake of DiL-TDNPs 2 was energy-dependent. TDNPs 2 were preferentially localized to the inflamed colon via oral administration and then internalized by colonic epithelial and macrophage cells. Energy-dependent caveolae-mediated endocytosis might be involved in the cellular uptake for TDNPs 2.

### Oral administration of TDNPs 2 alleviate DSS-induced colitis

**Fig. 6:**
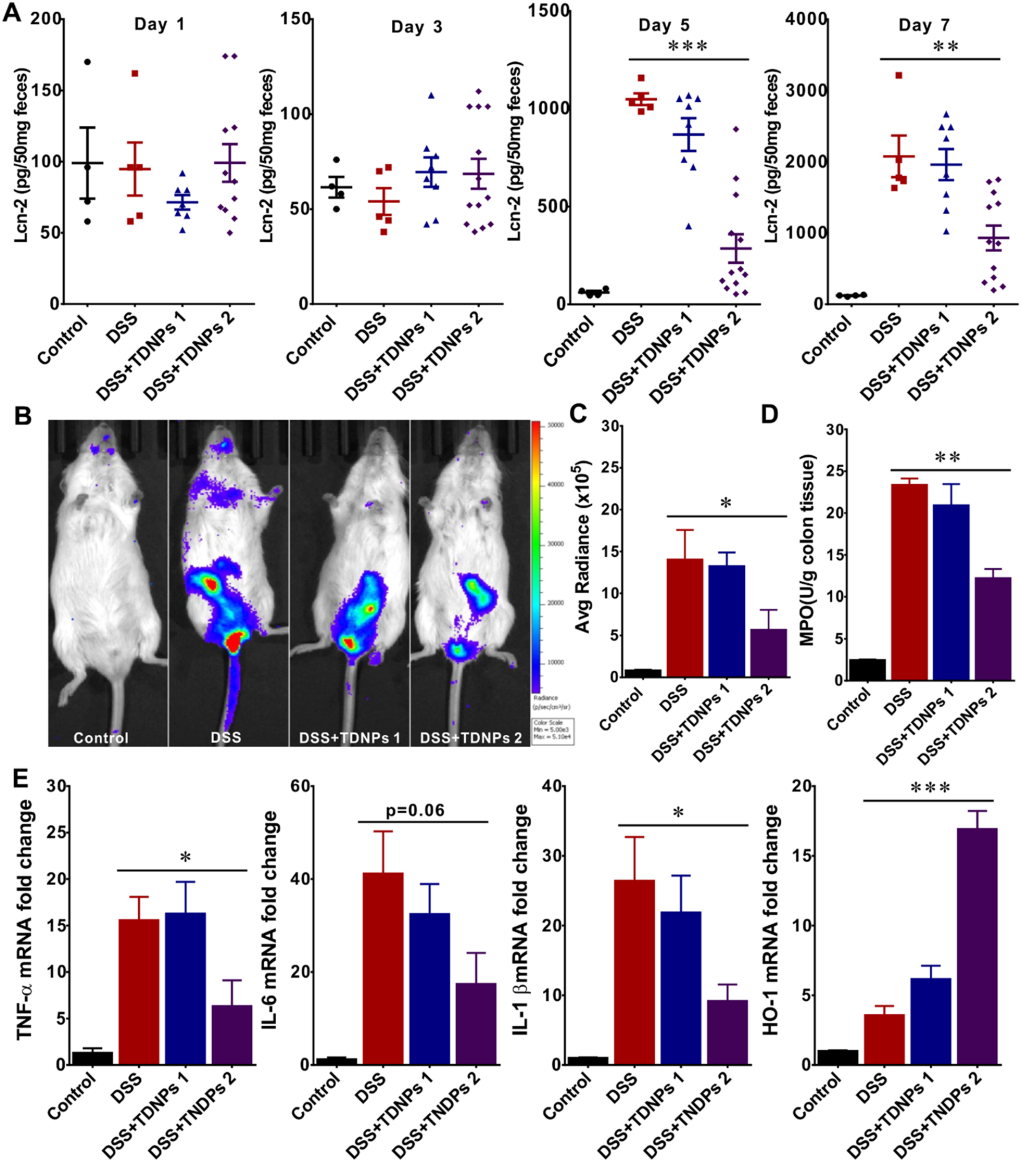
Oral administration of TDNPs 2 alleviated DSS-induced colitis. **A** Lipocalin-2 quantification (n = 5). **B** Colon inflammation was monitored by XenoLight RediJect inflammation probe via chemiluminescence imaging (n = 3). **C** Average radiance was captured and compared between groups (n = 5). **D** MPO quantification (n = 5). **E** Real-time PCR to quantify miRNA expression (n = 5), *p < 0.05, **p < 0.01, ***p < 0.001

Inspired by the above findings that TDNPs 2 were preferentially localized to the inflamed colon, we then aimed to investigate the effect of TDNPs 2 on DSS-induced colitis, a well-established mice model for studying human UC [56, 57]. Mice were randomly divided into four groups, control (plain water), DSS group (3% DSS water), TDNPs 1, and TDNPs 2 treated groups. Lcn-2, as an attractive biomarker of intestinal inflammation, was used to monitor the progression of intestinal inflammation. From D1-D3, the expression levels of Lcn-2 were very low for all the groups, indicating inconspicuous inflammation for all the mice. From Day 5, Lcn-2 expression was still in the basal level in the control group. In contrast, Lcn-2 in DDS- and TDNPs 1-treated groups was markedly raised with a similar tendency. Surprisingly, Lcn-2 in TDNPs 2-treated group was still at a lower level when compared with DSS or TDNPs 1-treated groups. These results indicated that TDNPs 2 could alleviate DSS-induced colitis. Colon inflammation in treated groups was also monitored by chemiluminescence imaging using a commercially available inflammation probe [58, 59]. As shown in Fig. 6B, the abdomen of the mice in the DDS- and TDNPs 1-treated group showed obvious bioluminescence signals, indicating severe intra-digestive inflammation. In contrast, although there were still bioluminescence signals in TDNPs 2-treated mice, the intensity was much lower than DDS- and TDNPs 1-treated mice. The intensity of bioluminescence signals in Fig. 6B was quantized and compared (Fig. 6C). To confirm this observation, myeloperoxidase (MPO) levels in the colon tissues were also evaluated, which were consistent with Lcn-2 and chemiluminescence imaging results, and MPO levels in TDNPs 2-treated mice were much lower than DDS- and TDNPs 1-treated mice (Fig. 6D). During UC development, the secretion of pro-inflammatory cytokines induced reactive oxygen species (ROS) production. ROS caused damage to the intestinal mucosa directly and induced oxidative stress in colon epithelial cells, resulting in a variety of lipid peroxidation. ROS also had a chemotactic ability. It recruited immune cells such as neutrophils into the site of inflammation to further stimulate the secretion of pro-inflammatory cytokines, thereby amplifying the intestinal inflammatory response and leading to increased damage [60–63]. Therefore, pro-inflammatory cytokines and oxidative stress played an important role in colitis. The expressions of pro-inflammatory cytokines (TNF-α, IL-6, and IL-1β) and oxidative stress-related protein, HO-1, were elevated in the DSS group, as compared, the levels of pro-inflammatory cytokines (TNF-α, IL-6, and IL-1β) were dramatically decreased, while oxidative stress-related HO-1 was significantly increased in TDNPs 2 treated group (Fig. 6E). Heme oxygenase-1 (HO-1) possesses many protective effects, which play a role in the physiological state and can be induced in abnormal conditions, including inflammation. Therefore, HO-1 is considered a critical factor in maintaining oxidative and antioxidant homeostasis in cells damaged [64, 65]. Our results demonstrated that TDNPs 2 could alleviate colitis with anti-inflammatory and antioxidant effects.

**Fig. 7:**
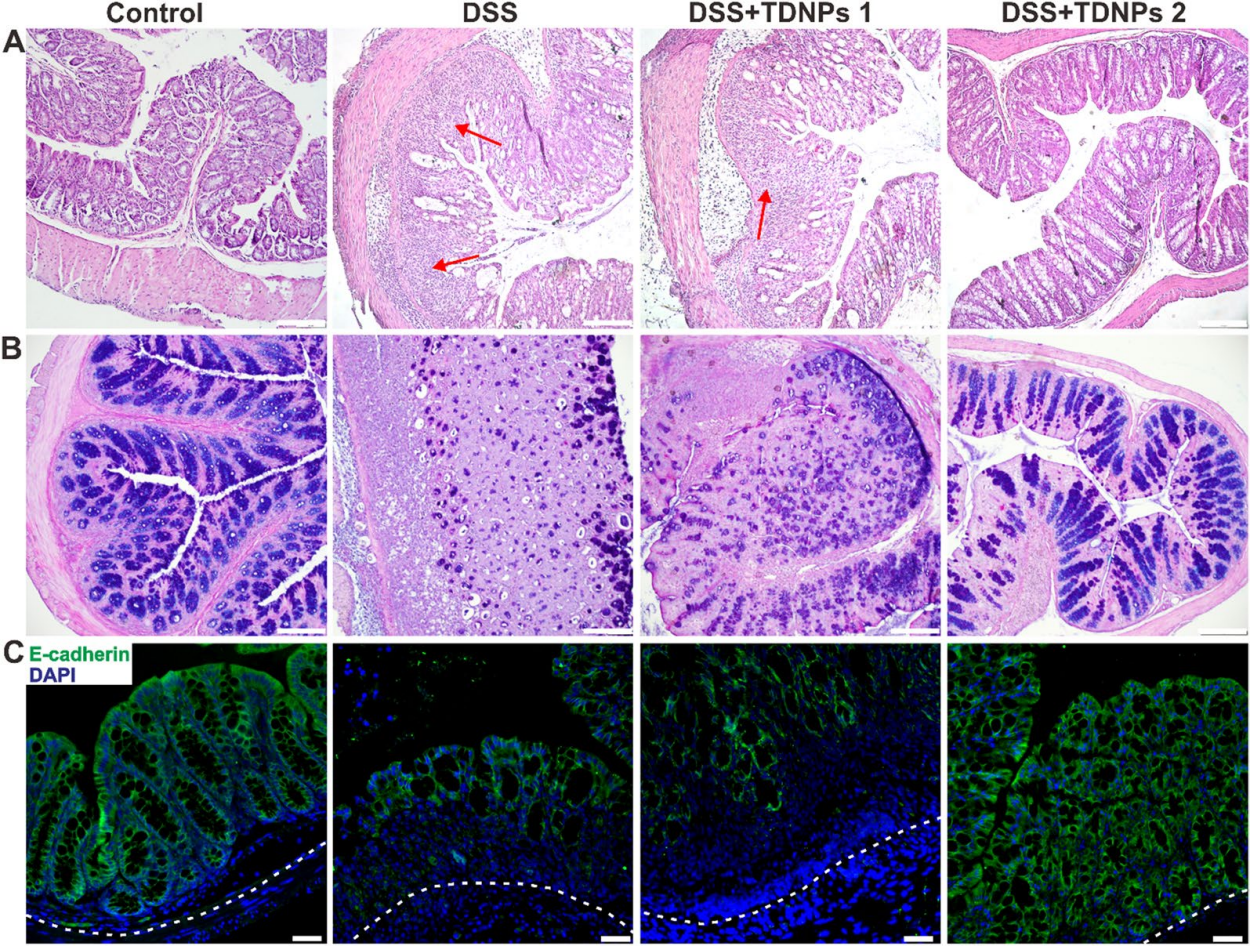
Histological stain to evaluate the protective effect of TDNPs 2 on colitis. **A** Representative H&E-stained colon. Inflammatory cell infiltration was indicated by arrowheads. **B** Colonic goblet cells were stained by Alcian blue. As goblet cells produce mucins, Alcian blue was used to highlight the goblet cell population. **C** E-cadherin expression was detected by immunofluorescence. Scale bar: 50 μm

The histological effects of TNDPs 2 on colitis were first evaluated by H&E staining (Fig. 7A). DDS- and TDNPs 1-treated mice exhibited robust signs of inflammation, including increased inflammatory cells infiltration in the lamina propria (indicated by arrowheads), epithelial erosion, and interstitial edema. In contrast, mice treated with TDNPs 2 showed decreased signs of inflammation at the histological level, including significantly reduced lymphocytic infiltration to lamina propria. Colonic goblet cells and their main secretory product, mucus, played essential roles in maintaining mucosal immunology and biology of the intestinal tract; for example, the mucus slows down bacterial penetration to push bacteria out toward the lumen. To highlight colonic goblet cells, alcian blue staining was used to identify acid mucin, neutral mucin, and glycogen. In DDS- and TDNPs 1-treated mice, colonic goblet cells were significantly damaged. When treated with TDNPs 2, the colonic goblet cells looked intact (Fig. 7B). E-cadherin is an important molecule involved in adhesion junctions between cells that maintain cell polarity and tissue structural integrity. E-cadherin’s abnormal expression was related to cell migration and invasiveness. Therefore, E-cadherin played an important role in maintaining epithelial cell polarity and regulating the permeability of the intestinal barrier [66–68]. In immunofluorescence results, E-cadherin on colonic epithelial cells in TDNPs 2 treated mice was highly expressed than DSS- and GDNPs 1-treated mice (Fig. 7C). These results corroborated the results of H&E staining and alcian blue staining.

### Oral administration of TDNPs 2 accelerate inflammation resolution of colitis

**Fig. 8:**
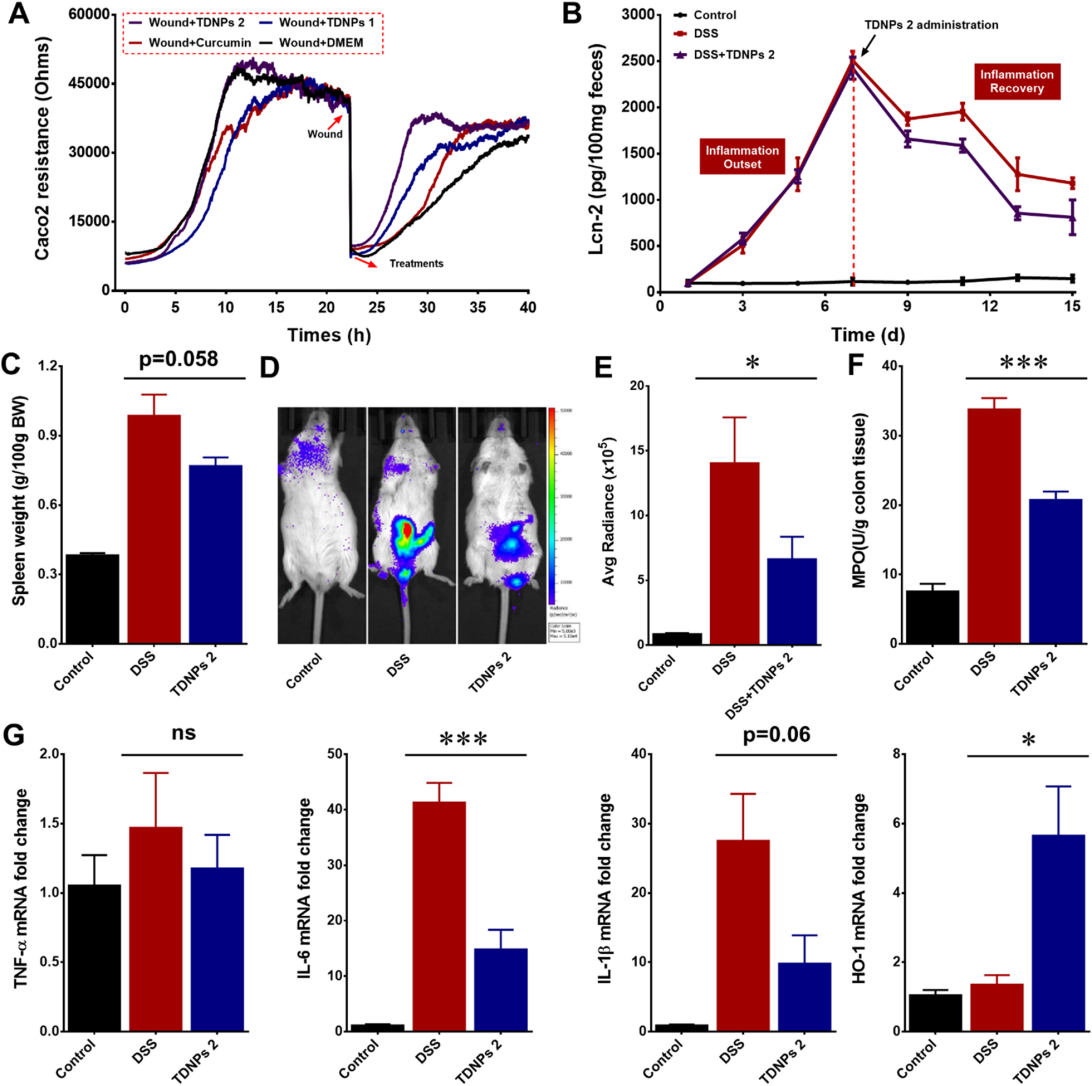
Oral administration of TDNPs 2 accelerated inflammation resolution of colitis. **A** ECIS wound healing assay. **B** Lcn-2 quantification (n = 5). **C** Spleen weight (n = 5). **D** Colon inflammation was monitored by XenoLight RediJect inflammation probe via chemiluminescence imaging. **E** Average radiance (n = 5). **F** MPO quantification (n = 5). **G** Real-time PCR to quantify miRNA expression (n = 5). *p < 0.05, ***p < 0.001 and ns represent no significant difference

We have confirmed that TDNPs 2 could alleviate DSS-induced colitis. We next evaluated whether TDNPs 2 could accelerate inflammation resolution of colitis. The electric cell-substrate impedance sensing (ECIS) was used to perform the wound-healing assay in vitro [69, 70]. Caco2 cells were cultured to form a monolayer in a tailor-made ECIS dish. When the impedance traces reached a plateau, an elevated electric field was applied to the wells to induce a wound of the cell, which caused a cliff-like drop in the impedance traces. DMEM (control), curcumin, TDNPs 1, or TDNPs 2 were added to the wells to assess their effects on cell wounds at the lowest impedance point. Results showed that TDNPs 2-treated cells had the fastest migration speed to healing the wound when compared with other groups, indicating TDNPs 2 accelerated wound repair of Caco2 cells (Fig. 8A). Colitis resolution experiments were also performed in vivo. As shown in Fig. 8B, Lcn-2 was used as a biomarker to monitor colitis development. In the inflammation outset phase, mice were drunk with DSS water to induce colitis (D1-D7). In the inflammation recovery phase (D7-D15), the DSS water was exchanged with plain water. In TDNPs 2-treated group, mice were orally administrated with TDNPs 2 to investigate its effect on colitis resolution. Lcn-2 levels gradually elevated from D1-D7 when DSS was present, suggesting that DSS successfully induced colitis. During the inflammation recovery phase, when treated with TDNPs 2, the resolution of inflammation was significantly accelerated compared with the control group. Spleen weight was correlated with the degree of inflammation [71]. The spleen weight between groups was compared at the end of the experiment. Spleen weight in TDNPs 2-treated group decreased significantly compared with the DSS group, indicating ameliorative inflammation by treating with TDNPs 2 (Fig. 8C). Colon inflammation was also monitored by chemiluminescence imaging using a commercially available inflammation probe. The bioluminescence signals in TDNPs 2 treated mice were weaker than those in DDS-treated mice (Fig. 8D and E). Myeloperoxidase (MPO) levels were consistent with the above-mentioned results. MPO levels in TDNPs 2 group were much lower than those in the DSS group (Fig. 8F). For mRNA expressions, pro-inflammatory cytokines (TNF-α, IL-6, and IL-1β) were decreased, while oxidative stress-related protein, HO-1, increased in TDNPs 2-treated mice compared with DDS-treated mice (Fig. 8G). Collectively, oral administration of TDNPs 2 appeared to accelerate colitis resolution.

### Biocompatibility evaluation of TDNPs 2

TDNPs 2 exerted excellent therapeutic outcomes to colitis with anti-inflammatory and pro-resolving bioactions. The biocompatibility of TDNPs 2 should be evaluated comprehensively to meet the potential clinical application [72, 73]. Firstly, an ECIS assay was performed to assess the toxicity of TDNPs 2 towards Caco2 cells for real-time measurement of cell proliferation (Additional file 1: Fig. S6A). The results showed that TDNPs 2 (up to 100 μg/ml) did not cause resistance change when compared with DMEM groups. In contrast, the positive control (Tween-100) had a steep decrease in resistance, indicating the excellent biocompatibility of TDNPs 2 in vitro. Other methods, including MTT assay (Additional file 1: Fig. S6B), ATPLite assay (Additional file 1: Fig. S6C), cell apoptosis assay (Additional file 1: Fig. S6D), and activated caspase-3/7 (Additional file 1: Fig. S7), also demonstrated the excellent biocompatibility of TDNPs 2.

**Fig. 9:**
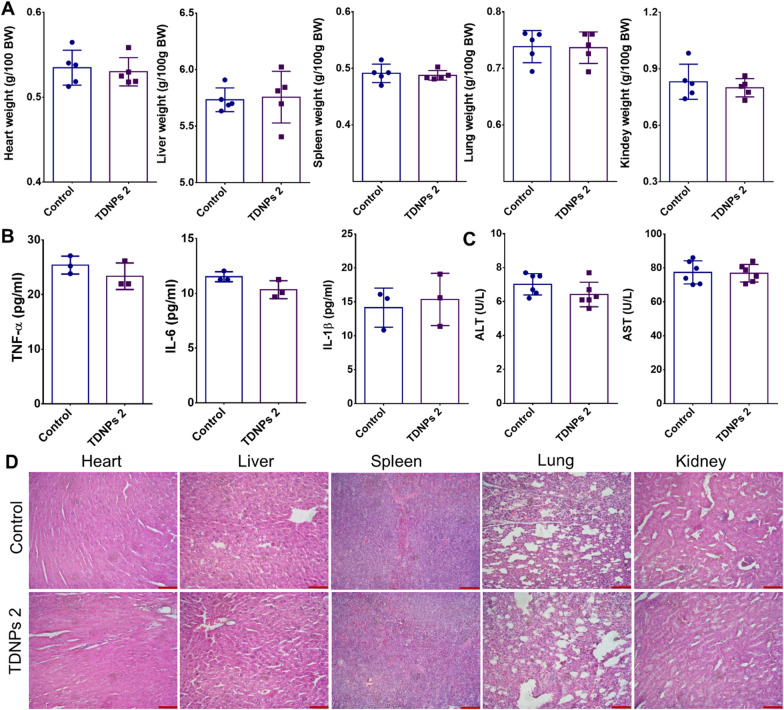
Biocompatibility evaluation of TDNPs 2. **A** Vital organs weights (n = 5). **B** Pro-inflammatory cytokines (n = 5). **C** Indicators reflected the physiological function of the liver were evaluated. **D** H&E staining, scale bar: 50 μm

Next, the biosafety of TDNPs 2 in vivo was explored. The weights of vital organs in the TDNPs 2-treated group were comparable with the control (Fig. 9A). Pro-inflammatory cytokines (IL-6, IL-1β, and TNF-α) in serum did not significantly change between TDNPs 2 and control groups (Fig. 9B). ALT and AST, which reflect the functions of the liver, also seemed normal between these groups (Fig. 9C). H&E staining for histological analysis of vital organs did not find any clear evidence of organ damage in TDNPs 2-treated mice compared with control mice (Fig. 9D). Since TDNPs 2 are isolated from edible turmeric, they should have excellent biocompatibility, and our comprehensive evaluation provides experimental evidence.

### TDNPs 2 exert a protective effect by inactivating NF-κB pathway

**Fig. 10:**
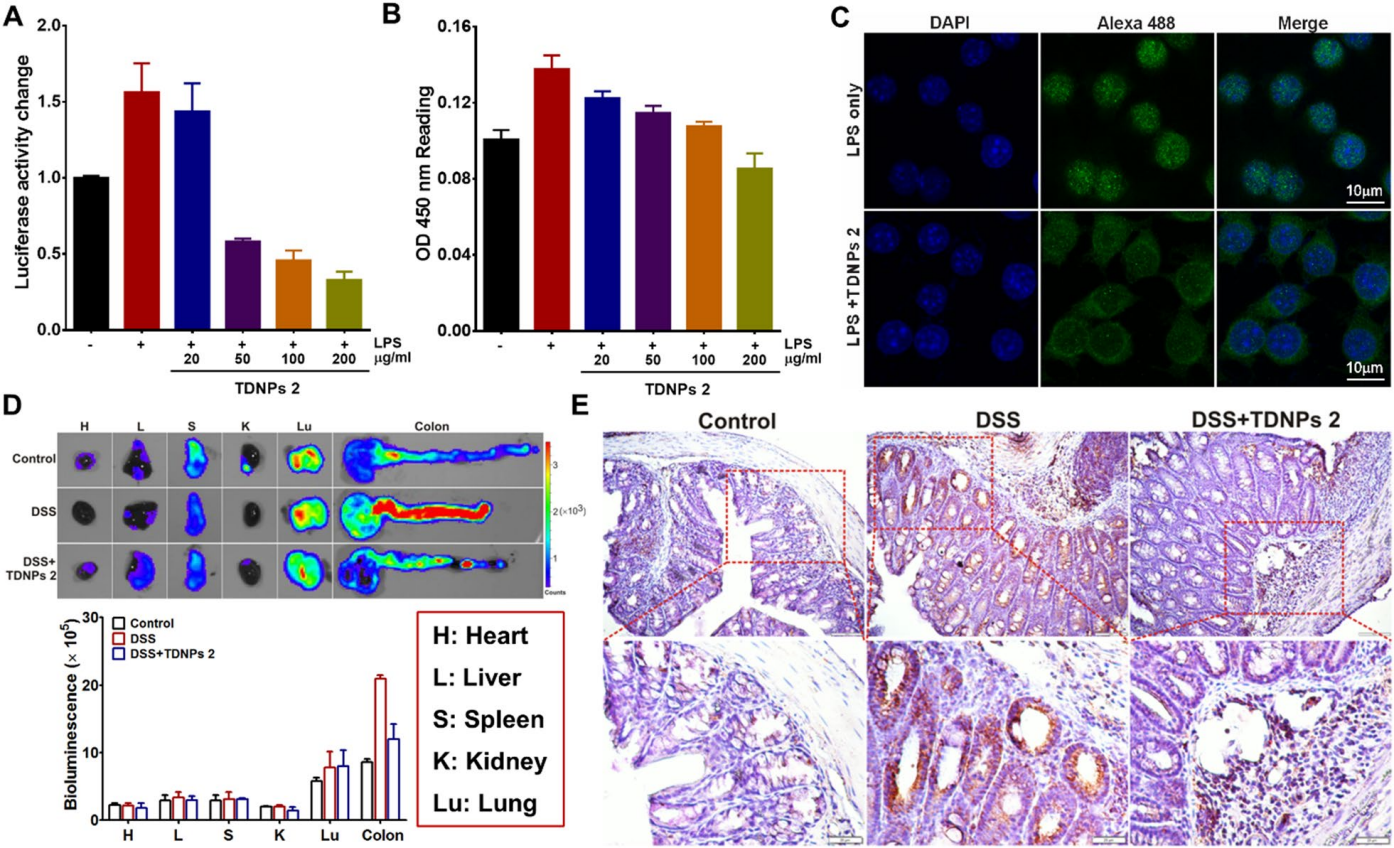
TDNPs 2 exerted a protective effect by inactivating the NF-κB pathway. **A** NF-κB activity evaluation (n = 5). **B** Phospho-NF-κB p65 expression was evaluated by ELISA assay (n = 5). **C** The translocation of NF-kB-p65 to the nucleus was assessed by immunofluorescence, scale bar = 10 μm. **D** NF-κB-RE-Luc transgenic mice was used to investigate NF-κB activity in vivo (n = 5). **E** NF-κB expression in the colon was evaluated by IHC

Nuclear factor-kappa B (NF-κB) is an important nuclear transcription factor that plays an essential role in regulating the inflammatory response. Curcumin was an NF-κB inhibitor and exhibited extensive properties [74, 75]. Given this information, we aimed to investigate whether TDNPs 2 could affect the NF-kB pathway. To verify our hypothesis, RAW 264.7 cells were co-transfected with the luciferase reporter, which was introduced in detail in the previous work [76]. RAW 264.7 cells were then treated with LPS in the presence of TDNPs 2 (20 ~ 200 μg/ml). The results showed LPS could induce NF-κB-dependent luciferase reporter expression (~ 1.6 fold increase), when treated with TDNPs 2, the luciferase activity was suppressed in a concentration-dependent manner (Fig. 10A). ELISA assay for phospho-NF-κB p65 was also revealed that TDNPs 2 suppressed phospho-NF-κB p65 expression, and it was consistent with the luciferase reporter activity experiments (Fig. 10B). Confocal imaging confirmed that TDNPs 2 could affect the translocation of NF-κB-p65 to the nucleus (Fig. 10C). Thus, activation of the NF-κB pathway by LPS was inhibited by TDNPs 2, as demonstrated by measuring NF-κB-p65-dependent luciferase activity, phospho-NF-κB p65 expression and translocation of p65 to the nucleus. To investigate the inhibition of TDNPs 2 to the NF-κB pathway in vivo, NF-κB-RE-Luc transgenic mice were used to explore NF-κB activity. At the end of the experiment, vital organs (heart, liver, spleen, kidney, and lung) and colon were captured and imaged. As shown in Fig. 10D, the bioluminescence signals of vital organs were similar, indicating similar NF-κB activities in these organs. By contrast, the bioluminescence signal of the colon in TDNPs 2 treated groups was obviously decreased compared with DSS. Immunohistochemistry stain of NF-κB was also confirmed the decreased NF-κB expression in TDNPs 2 treated colon tissue (Fig. 10E). Collectively, these results demonstrated that TDNPs 2 exerted a protective effect by inactivating the NF-κB pathway.

## Discussion

Nanoparticulate systems have attracted attention for colitis treatment, as they have unique physicochemical properties and disease-site targeting capabilities. Various strategies are currently being investigated, including ligand/receptor, charge, size, degradation, and microbiome-mediated delivery strategies [77, 78]. Nanovesicles extracted from plants are deemed to be a branch of nanomedicine. Plant-derived nanovesicles possess the function to communicate between the plant and animal kingdoms and thus have the therapeutic potential against various diseases, including UC [79].

In the current study, two populations of TNDPs (TDNPs 1 and TDNPs 2) from edible turmeric were identified. Both of them are nano-sized with negative zeta potential. In UC, inflammation of the colonic mucosa is well known to be accompanied by depletion of the mucus layer and in situ accumulation of positively charged proteins, including transferrin, anti-microbial peptides, and bactericidal/permeability-increasing proteins. Accumulation of these proteins results in a positive charge at the damaged epithelial surface, providing an anchor for negatively charged drug carriers [80]. TDNPs 2 with remarkable targeting ability to the inflamed colon may be at least partly attributed to their electrostatic interaction with the damaged epithelial surface. Lipid profiling revealed that the TDNPs predominantly comprised digalactosyldiacylglycerol (DGDG), monogalactosyl diacylglycerol (MGDG), phosphatidylinositol (PI), phosphatidylcholine (PC), and phosphatidic acid (PA), which were present in different amounts in TDNPs 1 and TDNPs 2. PC was approved to have a protective function to colonic mucus among these components. The lower intrinsic mucus PC content in UC patients affects the intestinal mucus with a hydrophobic barrier function. This is thought to allow colonic bacteria to permeate the intestinal mucus barrier, leading to nonspecific but aggressive immune responses followed by inflammation and ulceration [81]. Therefore, our findings suggest that TDNPs could be a potential natural turmeric-derived targeted therapeutics.

Compared to TDNPs 1, TDNPs 2 had many more proteins and were enriched for cytosolic and membrane proteins. Many of the proteins in TDNPs 2 were found to represent uncharacterized proteins, indicating the undiscovered functions and relevance with TDNPs 2 or turmeric and needed to lucubrate. Interestingly, TDNPs 2 contained a higher curcumin content, the main active ingredient of turmeric, compared to TDNPs 1. The density of TNDPs 2 in sucrose (1.13–1.19 g/ml) was similar to that of exosomes from mammalian cells. Based on these findings, we defined TDNPs 2 as turmeric-derived exosome-like nanoparticles and used them for further experiments.

Conventional therapeutic drugs for UC are limited in clinical use. They tend to be administrated via intravenous (*i.v.*) injection or other systemic routes, leading to toxicity and adverse effects with the systemic distribution of the drug. In contrast, TDNPs 2 were delivered orally, which offered incomparable advantages, such as fewer adverse reactions, does not directly damage the skin or mucous membranes, economical and convenient for patients. Indeed, our data demonstrated that orally administrated TDNPs 2 preferentially localized to the inflamed colon. This is consistent with previous studies in which grape- and ginger-derived exosome-like nanoparticles targeted the colon [82]. TDNPs 2 are efficiently internalized by colonic epithelial and macrophage cells upon reaching the inflamed colon. This dual cellular targeting ability of TDNPs 2 contrasts with the abilities of nanoparticles from grape or grapefruit, which primarily targeted intestinal macrophages or intestinal stem cells (ISCs), respectively [83].

In the DSS-induced colitis model, TDNPs 2 relieved acute inflammation and reduced the colitis susceptibility of mice. The acute inflammatory response involves a complex but highly coordinated sequence of events, including molecular, cellular, and physiological alterations [84]. Acute inflammation begins with producing soluble mediators (including complements, chemokines, cytokines, free radicals, and vasoactive amines) by endothelial cells, macrophages, and other resident cells of the injured or infected tissue. After being internalized by endothelial cells and macrophages, TDNPs 2 appeared to impact the inflammatory state: Our results showed that TDNPs 2 suppressed the expressions of pro-inflammatory cytokines (TNF-α, IL-6, and IL-1β) while increasing the expression of an antioxidant gene (heme oxygenase 1, HO-1), suggesting that TDNPs 2 have the potential to attenuate damaging factors and promote protective factors.

Notably, TDNPs 2 also promoted the resolution of colitis. Inflammation resolution stage is the period between the peak inflammatory cell influx and the clearance of inflammatory cells from the tissue site/restoration of functional homeostasis. This occurs via a complex and tightly regulated cascade of processes [85]. Interestingly, we found that treatment of wounded intestinal mucosal with TDNPs 2 recovered the normal levels of anti-inflammatory cytokines and MPO activity. Our results suggest that TDNPs 2 have both anti-inflammation and pro-resolution functions and thus might offer a therapeutic strategy that is superior to the existing approaches.

In conclusion, we present a novel, natural, and nontoxic exosome-like nanovesicle that can preferentially localize to the inflamed colon and possesses anti-inflammation and pro-resolution functions. This system, exemplified by TDNPs 2, can easily be developed for large-scale production and may represent an effective therapeutic strategy against UC.

## Supplementary Information

**Additional file 1. Figure S1.** Size and zeta potential of turmeric-derived nanoparticles (TDNPs). **Figure S2.** Evaluation of the bioactive compound, curcumin, in turmeric juice and TNDPs by HPLC-UV. (A) Analytical curve of standard curcumin, TNDPs 1 and TDNPs 2. (B) Curcumin content was normalized, n=3. **Figure S3.** Stability investigation of TDNPs. **Figure S4.** Investigation the potential endocytosis pathway of TDNPs 2. Special inhibitors were incubated with RAW 264.7 cells, then DiL-TDNPs 2 were added. Finally, cells were coverslip-mounted with DAPI for confocal imaging, scale bar: 20 µm. **Figure S5.** Investigation the effect of temperature on TDNPs 2 internalization. DiL-TDNPs 2 were incubated with RAW 264.7 cells under different temperatures, then cells were stained and imaged be confocal microscopy, scale bar: 20 µm. **Figure S6.** Assess the biocompatibility of TDNPs 2 in vitro. (A) Real-time in vitro cellular cytotoxicity of TNNPs 2 on Caco2 cells was monitored using electric cell-substrate impedance sensing (ECIS). (B) MTT assay was used to assess the potential toxicity of TDNPs 2 in macrophage 264.7 cells and colon-26 cells, (n=5). (C) Macrophage 264.7 and colon-26 cells proliferations were evaluated by ATPlite assays, (n=5). (D) Apoptosis of macrophage 264.7 and colon-26 cells was determined by Annexin V/PI stain assay, n=5. **Figure S7.** Apoptosis of macrophage 264.7 and colon-26 cells was determined by the presence of activated caspase-3/7. Apoptotic cells (green nuclei) were detected by staining for cleaved caspase-3/7, scale bar: 20 µm. **Table S1. **Lipids found in Turmeric-derived nanoparticles. **Table S2.** Proteomics analysis of TDNPs 2. **Table S3.** Primers used for Real-time PCR.

## Data Availability

All data needed to support the conclusions are present in the paper and/or the Supplementary Materials. Additional data related to this paper may be requested from the authors.
