# Supplementary material for: Oral administration of turmeric-derived exosome-like nanovesicles with anti-inflammatory and pro-resolving bioactions for murine colitis therapy
Source: J Nanobiotechnology. 2022 Apr 29;20:206. doi: 10.1186/s12951-022-01421-w (PMC9052603; doi:10.1186/s12951-022-01421-w)
Supplement: Supplementary file 1 — Additional file 1. Figure S1. Size and zeta potential of turmeric-derived nanoparticles (TDNPs). Figure S2. Evaluation of the bioactive compound, curcumin, in turmeric juice and TNDPs by HPLC-UV. (A) Analytical curve of standard curcumin, TNDPs 1 and TDNPs 2. (B) Curcumin content was normalized, n=3. Figure S3. Stability investigation of TDNPs. Figure S4. Investigation the potential endocytosis pathway of TDNPs 2. Special inhibitors were incubated with RAW 264.7 cells, then DiL-TDNPs 2 were added. Finally, cells were coverslip-mounted with DAPI for confocal imaging, scale bar: 20 µm. Figure S5. Investigation the effect of temperature on TDNPs 2 internalization. DiL-TDNPs 2 were incubated with RAW 264.7 cells under different temperatures, then cells were stained and imaged be confocal microscopy, scale bar: 20 µm. Figure S6. Assess the biocompatibility of TDNPs 2 in vitro. (A) Real-time in vitro cellular cytotoxicity of TNNPs 2 on Caco2 cells was monitored using electric cell-substrate impedance sensing (ECIS). (B) MTT assay was used to assess the potential toxicity of TDNPs 2 in macrophage 264.7 cells and colon-26 cells, (n=5). (C) Macrophage 264.7 and colon-26 cells proliferations were evaluated by ATPlite assays, (n=5). (D) Apoptosis of macrophage 264.7 and colon-26 cells was determined by Annexin V/PI stain assay, n=5. Figure S7. Apoptosis of macrophage 264.7 and colon-26 cells was determined by the presence of activated caspase-3/7. Apoptotic cells (green nuclei) were detected by staining for cleaved caspase-3/7, scale bar: 20 µm. Table S1. Lipids found in Turmeric-derived nanoparticles. Table S2. Proteomics analysis of TDNPs 2. Table S3. Primers used for Real-time PCR. [file 12951_2022_1421_MOESM1_ESM.docx]

**Supporting information**

**Oral administration of turmeric-derived exosome-like nanovesicles with anti-inflammatory and pro-resolving bioactions for murine colitis therapy**

Cui Liu^1,2#^, Xiangji Yan^1,2#^, Yujie Zhang^1,2#^, Mei Yang^1,2^, Yana Ma^1,2^, Yuanyuan Zhang^1,2^, Qiuran Xu^3*^, Kangsheng Tu^4*^, Mingzhen Zhang^1,2,5*^

Affiliations:

1. School of Basic Medical Sciences, Xi'an Key Laboratory of Immune Related Diseases, Xi'an Jiaotong University, Xi'an, Shaanxi, 710061, China
2. Key Laboratory of Environment and Genes Related to Diseases, Xi'an Jiaotong University, Ministry of Education, Xi'an, Shaanxi, 710061, China
3. Laboratory of Tumor Molecular Diagnosis and Individualized Medicine of Zhejiang Province, Zhejiang Provincial People's Hospital, Affiliated People's Hospital, Hangzhou Medical College, Hangzhou, Zhejiang, 310014, China
4. Department of Hepatobiliary Surgery, the First Affiliated Hospital of Xi'an Jiaotong University, Xi'an, Shaanxi, 710061, China
5. Institute for Biomedical Sciences, Center for Diagnostics and Therapeutics, Digestive Disease Research Group, Georgia State University, Atlanta, Georgia, 30302, United States.

^#^ These authors contributed equally to this article.

**Supplementary methods**

**Chemicals**

Phalloidin-FITC, O-dianisidine dihydrochloride, myeloperoxidase standard from human leukocytes, type VIII collagenase, DNase I, curcumin standard, ALT and AST assay kits, TUNEL in situ cell death detection kit, and pathway inhibitors (cytochalasin D, amiloride, indomethacin and chlorpromazine) were purchased from Sigma (St. Louis, MO, USA); Fluorescent lipophilic dyes, 1,1'-dioctadecyl-3,3,3',3'-tetramethylindocarbocyanine perchlorate (DiL), 3,3'-dioctadecyloxacarbocyanine perchlorate (DiO) and 1,1'-dioctadecyl-3,3',3'- tetramethylindotricarbocyanine iodide (DiR) were purchased from Promokine (Heidelberg, Germany); Rabbit anti-mouse E-cadherin antibody was from Santa Cruz Biotechnology (Santa Cruz, CA, USA); Anti-mouse CD326 (EpCAM) PE-Cy7, anti-mouse CD11b-eFluo 450, anti-mouse CD11c-APC and anti-mouse F4/80 antigen PE-Cy7 were purchased from eBioscience (San Diego, CA, USA). Duoset enzyme-linked immunosorbent assay (ELISA) kits were purchased from R&D Systems (Minneapolis, MN, USA). ATP determination kit, CellEvent™ Caspase-3/7 Green Detection Reagent, Vybrant® MTT cell proliferation assay kit, and Annexin V-FITC/propidium iodide (PI) apoptosis detection kit were obtained from ThermoFisher Scientific (Eugene, OR, USA).

**Isolation, purification, and characterization of turmeric-derived nanoparticles (TDNPs)**

Turmeric or *Curcuma Longa* (Order, Zingiberales; Family, Zingiberaceae; Genus, Curcuma) was purchased from a local farmer's market, which was used to isolate TDNPs. According to the protocol developed in our lab, turmeric was washed thoroughly with tap water at room temperature (22 °C). After the final washing, the turmeric was ground in a Breville extractor to obtain juice. Then the juice was centrifuged first at 3000*g* for 20 min and then at 10 000*g* for 40 min to remove large fibers. The supernatant was ultra-centrifuged at 150,000*g* for two h, and the final obtained pellet was suspended in phosphate-buffered saline (PBS) through ultrasonic dispersion.

For TDNPs purification, the suspension of TNDPs was transferred to a discontinuous sucrose gradient (8%, 30%, and 45% [g/v]) and ultra-centrifuged at 150,000*g* for an additional two h. The bands between 8/30% and 30/45% layers, corresponding to TDNPs 1 and TDNPs 2, were harvested separately. The concentrations of obtained TDNPs thus were quantified based on protein concentration using a Bio-Rad protein quantification assay kit. The quantified TDNPs were stored at -80°C for further use.

TDNPs were characterized with respect to size and zeta potential by dynamic light scattering using a Zetasizer Nano ZS (Malvern, Southborough, MA). Atomic force microscopy (AFM) images were acquired using a SPA 400 AFM instrument (Seiko Instruments Inc., Chiba, Japan). For transmission electron microscopy (TEM) imaging, a drop of the sample was deposited onto the surface of a formvar-coated copper grid, after which 1% uranyl acetate was added for 15 s, and the sample was allowed to dry at room temperature for subsequent imaging.

For *in vitro* stability tests, 1.34 μl of 18.5% (w/v) HCl (pH 2.0) and 24 μl of pepsin solution (80 mg/ml in 0.1 N HCl, pH 2.0) were added to 1 ml (1 mg/ml) of TDNPs in PBS, and the mixture was incubated at 37 °C for 0.5 h (stomach-like conditions). Then, 80 μl of a mixture containing 24 mg/ml of bile extract and 4 mg/ml of pancreatin in 0.1 N NaHCO_3_ was added. The pH was adjusted to 6.5 with 1 N NaHCO_3_ and incubated for an additional 0.5 h under the same conditions (Small intestine-like conditions). The stability of TDNPs was evaluated by measuring particle size and zeta potential using the method described above.

**Lipids composition and proteomics profile analysis of TDNPs**

For TDNPs lipids composition analysis, lipid samples extracted from TDNPs 1 and TDNPs 2 (from discontinuous sucrose gradient) were submitted to the Lipidomics Research Center, Kansas State University (Manhattan, KS, USA) for analysis. Briefly, the lipid composition of TDNPs was determined using a triple quadrupole mass spectrometer (Applied Biosystems Q-TRAP; Applied Biosystems, Foster City, CA, USA), as described in an online protocol (http://www.k-state.edu/lipid/lipidomics/profiling.htm). Data for each lipid molecular species were presented as mol % of the total lipids analyzed.

For proteomics analysis, TDNPs 1 and TDNPs 2 purified from discontinuous sucrose gradient was shipped to Bioproximity on dry ice (Chantilly, VA, USA). Proteins in TDNPs 1 and TDNPs 2 were identified and quantified in a few hours from a few micrograms of protein by UPLC-MS/MS (ultra-performance liquid chromatography-tandem mass-spectrometry) using Orbitrap mass spectrometry.

**High-performance liquid chromatography with ultraviolet (HPLC-UV) spectrophotometer detection**

The HPLC system was comprised of a Shimadzu (VP series, Kyoto, Japan) pump (LC-20AT) with solvent cabinet, a degasser (DGU-20A3), a column oven (CTO-10S), an auto-injector (SIL-20A HT), UV/VIS detector (SPD-20AD) and computer software (LC-solution). The separation was carried out using a reversed-phase C-18 Agilent Eclipse Plus (Agilent, USA) column (250 × 4.6 mm ID, 5 μm). The flow rate was set at 1.5 mL/min, and the detection wavelength was 370 nm. Curcumin standard sample of 25 μl was injected onto the column.

**TDNPs labeling**

TDNPs were labeled with fluorescent lipophilic dyes, DiL, DIO, or DiR, depending on the experiment. Generally, 10 μM fluorescent dye solution was added to 1 mg TDNPs (1 ml in PBS), and the mixture was incubated for 30 min at room temperature. The labeled TDNPs were separated from the free dye by ultra-centrifuge.

**In vitro cellular uptake of TDNPs and potential endocytosis pathway(s) investigation**

For cellular uptake of TDNPs, RAW 264.7 microphage and Colon-26 cells were seeded in 8-chamber glass tissue culture slides (BD Falcon, Bedford, MA, USA) at a density of 1 × 10^5^ cells/well and incubated overnight in a growth medium. TDNPs 2 were labeled with DiL (Ex: 549 nm; Em: 565 nm) at a concentration of 10 μM. Subsequently, labeled TDNPs 2 (100 μg/ml) were incubated with cells for 4 h. After incubation, cells were fixed with 4% paraformaldehyde (PFA) for 15 min and then dehydrated with acetone at -20°C for 5 min. After blocking with 1 % bovine serum albumin (BSA) in PBS for 30 min, 100 μl of phalloidin-FITC (1:40 dilution) was added, and the mixture was incubated for an additional 30 min. Finally, cells were coverslip-mounted with a mounting medium containing 4-,6-diamidino-2-phenylindole (DAPI, H-1500; Vector Laboratories, Burlingame, CA, USA). Cells were observed and imaged using a Zeiss LSM 700 confocal microscope with Zen 2014 software version 9.1

To investigate the potential endocytosis pathway (s), special inhibitors (amiloride (250 μM), indomethacin (100 μM), chlorpromazine (25 μM) and cytochalasin D (10 μM)) were first incubated with RAW 264.7 cells for 1 h at 37°C, then 100 μg/ml of DiL labeled TDNPs 2 were added for additional 4 h incubation. After washing with 3 times PBS, cells were fixed with 4 % PFA for 15min. Finally, cells were coverslip-mounted with DAPI for fluorescence imaging.

To determine the effects of temperature on TDNPs internalization, RAW 264.7 cells were incubated with 100 μg/ml of DiL labeled TDNPs 2 for 4 h at 37°C, 20°C and 4°C, After washing with 3 times PBS, cells were fixed with 4 % PFA for 15min. Finally, cells were coverslip-mounted with DAPI for fluorescence imaging.

**Biocompatibility of TNDPs 2 both *in vitro* and *in vivo***

To test the biocompatibility of TDNPs 2 *in vitro*, Cell-attached assays were first performed to investigate the real-time cytotoxicity using electrical impedance sensing (ECIS) technology (Applied BioPhysics, Troy, NY, UAS), which is based on AC impedance measurements using weak and noninvasive AC signals. The attachment and spread of cells on the electrode surface change the impedance in such a way that morphological information about attached cells can be inferred. The measurement system consists of an 8-well culture dish (ECIS 8W1E plate) with the surface treated for cell culture. The bottom of each well contains a small, active electrode and a large counter electrode. A lock-in amplifier with an internal oscillator is used to switch among the different wells, and a personal computer controls the measurement and stores the data. For the experiment, Caco2-BBE cells at a density of 2×10^5^/well were seeded in the plate. Once cells reached confluence, TDNPs 2 with different concentrations (1, 10, 20, 50 and 100 μg/ml) and positive control, 1% Triton 100 were added to the wells. Basal resistance measurements were performed using the ideal frequency for Caco2-BBE cells, 500Hz, and a voltage of 1V.

For the MTT assay, RAW 264.7 and colon-26 cells were seeded in 96-well plates at a density of 1×10^4^ cells/well and incubated overnight. Cells were then incubated with different amounts of TDNPs 2 (1, 10, 20, 50 and 100 μg/ml) for 24 h. After TDNPs 2-containing medium was removed and cells were thoroughly rinsed once with PBS. Cells were then incubated with 20 μL of MTT (5 mg/ml) at 37°C for 4 h until a purple precipitate was visible. Thereafter, the media were discarded, and 50μl dimethyl sulfoxide (DMSO) was added to each well prior to spectrophotometric measurements at 570 nm. Untreated cells were used as a negative control.

For the ATPLite assay, RAW 264.7 and colon-26 cells were seeded in 96-well plates at a density of 1×10^4^ cells/well and incubated overnight. Cells were then incubated with with different amounts of TDNPs 2 (1, 10, 20, 50, and 100 μg/ml) for 24 h. The percentage of viable cells were then determined using the ATPLite assay following the protocol provided by the manufacturer. All experiments were conducted in triplicates.

For FACS assay to investigate the biocompatibility of TDNPs 2, RAW 264.7 and colon-26 cells were incubated with different amounts of TDNPs 2 (1, 10, 20, 50 and 100 μg/ml) for 24 h. Then cells were harvest and stained with Annexin V-FITC and PI and analyzed with a FACS Canto flow cytometer (BD Biosciences). Healthy cells were double negative in annexin V and PI staining, and early apoptotic cells were positive for annexin V but negative for PI staining. Necrotic cells were positive only for the PI, while late apoptotic cells were double positive. Excitation wave was set at 488 nm, and the emitted green fluorescence of annexin V and red fluorescence of PI were collected using 525 and 575 nm bandpass filters, respectively. Triplicate samples were analyzed for each experiment.

For caspase-3/7 detection, RAW 264.7 and colon-26 cells were incubated with TDNPs 2 (100 μg/ml) and positive control (doxorubicin, Dox) for 24 h. Then detection reagent (5 μM) were added and incubated for 30 min at 37 °C. Cells were then followed by fixation with 3.7 % formaldehyde for 15 min at RT. Finally, mounted with ProLong Gold reagent and cured for 24 h at RT in the dark before imaging. Apoptotic cells with activated caspase-3/7 show bright green nuclei, while cells without activated caspase-3/7 exhibit minimal fluorescence signals. The excitation/emission maxima for the CellEvent™ Caspase-3/7 Green Detection Reagent are 502/530.

**monitoring of inflammation during colitis *In vivo***

Chemiluminescence imaging was done using IVIS series preclinical in vivo imaging systems. Images of mice per group were captured and analyzed with Living Image® software (Perkin Elmer; Waltham, MA, USA). The inflammation of UC mice was assessed with the bioluminescent XenoLight RediJect Inflammation Probe, which is offered in a ready-to-use format and can be conveniently applied to study the myeloperoxidase (MPO) activity of activated phagocytes. Briefly, the abdominal region of the mice was softly shaved to reduce the absorption of light. Inflammation probe (150 μl/mouse, 40 mg/ml) was injected by IP 5 min before imaging.

**Quantification of Lipocalin-2 (Lcn-2)**

To quantify Lcn-2, freshly collected in mice feces were reconstituted in PBS containing 0.1% Tween 20 (50 mg/ml) and vortexed for 20 min to yield a homogeneous fecal suspension. Samples were then micro-centrifuged for 10 min at 4°C at full speed, and supernatants were collected for analysis (1: 20 dilution). Levels of Lcn-2 were estimated using a Duoset mouse Lcn-2 ELISA kit (R&D Systems).

**Histological analyses of tissue sections by hematoxylin and eosin staining**

Mice colons and different organs were fixed in 10 % formalin for 24 h or longer according to the tissue at room temperature, then embedded in paraffin. Tissues were sectioned at 6 μm thickness and stained with hematoxylin and eosin (H&E) using standard protocols established in our lab. Images were acquired using an Olympus microscope equipped with a DP-26 digital camera. Histological scores were assigned by experimenters "blinded" to sample identity. Colonic epithelial damage was assigned scores as follows: 0 = normal; 1 = hyperproliferation, irregular crypts, and goblet cell loss; 2 = mild to moderate crypt loss (10–50%); 3 = severe crypt loss (50–90%); 4 = complete crypt loss, surface epithelium intact; 5 = small- to medium-sized ulcer (<10 crypt widths); 6 = large ulcer (≥10 crypt widths). Infiltration with inflammatory cells was assigned scores separately for mucosa (0 = normal, 1 = mild, 2 = modest, 3 = severe), submucosa (0 = normal, 1 = mild to modest, 2 = severe), and muscle/serosa (0 = normal, 1 = moderate to severe). Scores for epithelial damage and inflammatory cell infiltration were added, resulting in a total scoring range of 0–12.

**NF-κB pathway investigation**

pGL4.32[luc2P/NF-κB-RE/Hygro] vector transfection experiment was used to determine NF-κB expression in cells. The pGL4.32[luc2P/NF-κB-RE/Hygro] Vector contains five copies of NF-κB response element (NF-κB-RE) that drives transcription of the luciferase reporter gene luc2P (Photinus pyralis). luc2P is a synthetically derived luciferase sequence with humanized codon optimization that is designed for high expression and reduced anomalous transcription. Briefly, RAW 264.7 cells were plated in 6-well plates. Covered the plate and placed plates in a tissue culture incubator at 37°C overnight. Followed, transfected the cells using a high-efficiency transfection reagent for 24h. Then, cells were co-treated with LPS (100 ng/ml) and different concentrations of TDNPs 2 (20, 50, 100 and 200 μg/ml). After 8h incubation, cells with different treatments were lysed, and luciferase activity was analyzed using an appropriate luciferase detection assay. The fold induction of can be calculated as follows: fold induction=average relative light units of induced cells/average relative light units of control cells.

Phospho-NF-κB p65 protein was also detected by CST's PathScan® Phospho-NF-κB p65 (Ser536) Sandwich ELISA Kit (#7173C). Briefly, RAW 264.7 cells were plated in 6-well plates overnight. Then, cells were co-treated with LPS (100 ng/ml) and different concentrations of TDNPs 2 (20, 50, 100 and 200 μg/ml). Finally, cells with different treatments were performed by PathScan® Sandwich ELISA Protocol. Absorbance at 450 nm was read and compared.

To detect of NF-κB-p65 complex by immunofluorescence, RAW 264.7 cells were seeded in glass-bottom cell culture chamber and cultured overnight. Cells were incubated with TDNP 2 (200μg/ml) or PBS and then induced by 100 ng/ml of LPS for 30 min. Followed, cells were fixed by paraformaldehyde (4%) and permeabilized by Triton X-100 (0.1%, v/v) for 10 min and 20 min, respectively. After blocking by BSA, cells were incubated with rabbit anti-NF-kB-p65 antibody (dilution 1:50; Cell Signaling # 8242) and anti-rabbit conjugated with Alexa 488 secondary antibody (dilution 1:50; Life Technologies). By staining the nucleus with DAPI, confocal microscopes were used to capture high-resolution photographs.

Real-time imaging of NF-κB activity *in vivo* was performed by NF-κB-RE-luc transgenic mice. Transgenic mice expressing modified firefly luciferase driven by six NF-κB-response-elements were developed by Caliper Life Sciences and obtained from Taconic (BALB/c-Tg(Rela-luc)31Xen). Mice transplanted with luciferase reporter cells were whole-body imaged for evidence of increased luciferase activity on a weekly basis by bioluminescence imaging (BLI). In the colitis model, transgenic mice were administrated with 3 % (w/v) DSS water and changed every other day. Mice were orally administered continuously with TDNPs 2 (3mg/dose) for 7 days. At the end of the experiment, mice were sacrificed and vital organs and colon were imaged by *in vivo* imaging system. During imaging, mice were anesthetized prior to injection of luciferin, and Luciferase transgene expression was measured. Optimal imaging occurred between 10 and 20 minutes, followed by intraperitoneal injection of luciferin. The expression of NF-κB in the colon was also evaluated by immunohistochemistry (IHC).

To evaluate the biocompatibility of TDNPs 2 *in vivo*, FVB mice were oral administration of TDNPs 2 for 14 day (3mg/day). After the last administration, mice were sacrificed, Body and different organ weights were measured at the same time. H&E staining was performed on paraffin-embedded heart, liver, spleen, lung, and kidney sections using the standard method. Pre-inflammatory cytokines levels in serum were measured by ELISA kits. ALT and AST in the sera of the mice were quantitatively analyzed using the commercially available kits from Sigma (ALT: MAK052; AST: MAK055).

**Anti-inflammatory and antioxidant effects of TDNPs in vitro**

To test the anti-inflammatory and antioxidant effects of TDNPs *in vitro*, RAW 264.7 cells were incubated with TDNPs 1 or TDNPs 2 (20, 50, 100, or 200 μg/ml) for 8 h, and then with LPS (10 ng/ml) for 1 h. Finally, Total RNA was extracted using an RNeasy Mini Kit (Qiagen, Valencia, CA, USA) according to the manufacturer's instructions. The yield and quality of extracted RNA were verified with a Synergy 2 plate reader (BioTek; Winooski, VT, USA). cDNA was generated from the total RNA isolated above using a Maxima First-Strand cDNA Synthesis kit (Thermo Scientific, Lafayette, CO, USA). Expression of target mRNAs was quantified by real-time reverse transcription-polymerase chain reaction (qRT-PCR) using Maxima SYBR green/ROX (6-carboxyl-X-rhodamine) qPCR Master Mix and primer pairs were shown in **Table S3**. Results were normalized by using 36B4 housekeeping gene.

**Supplementary Figures**


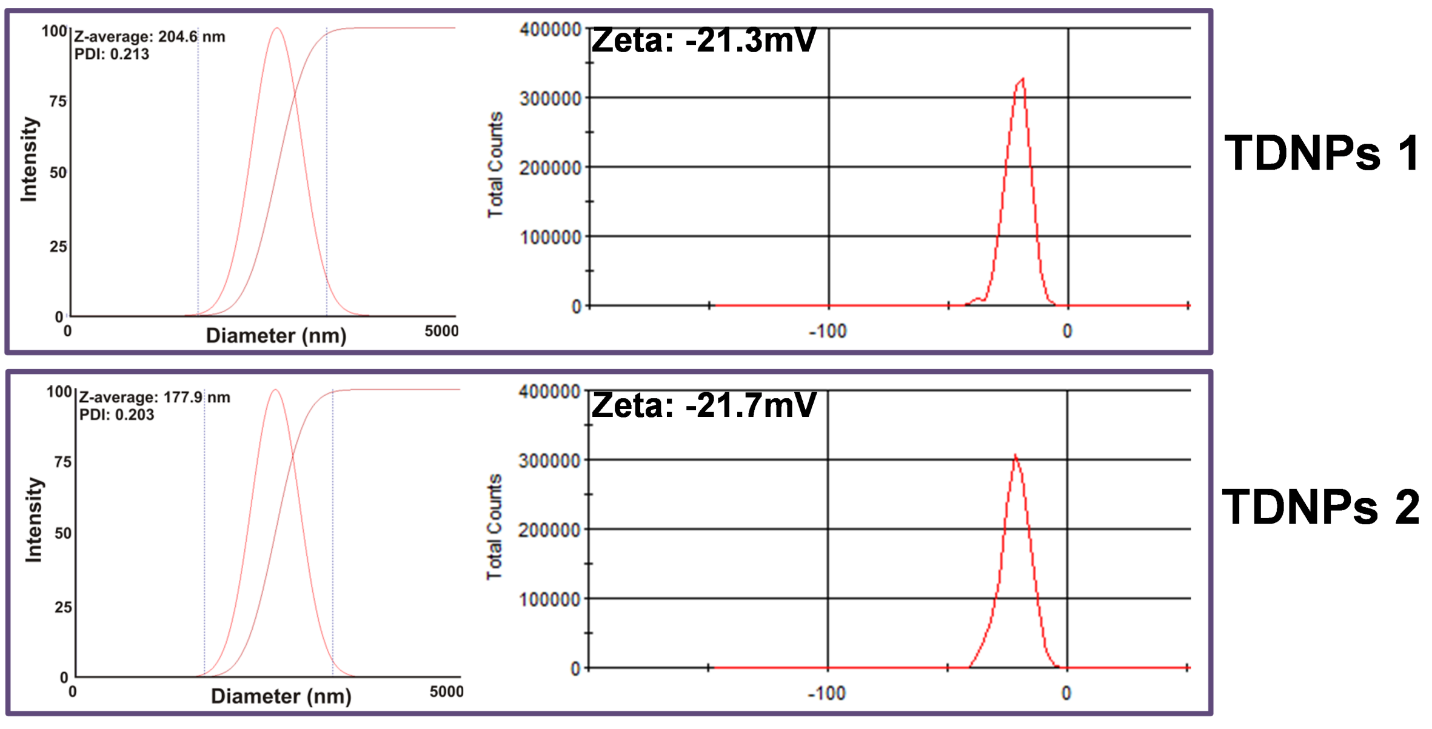


**Figure S1.** **Size and zeta potential of turmeric-derived nanoparticles (TDNPs).**


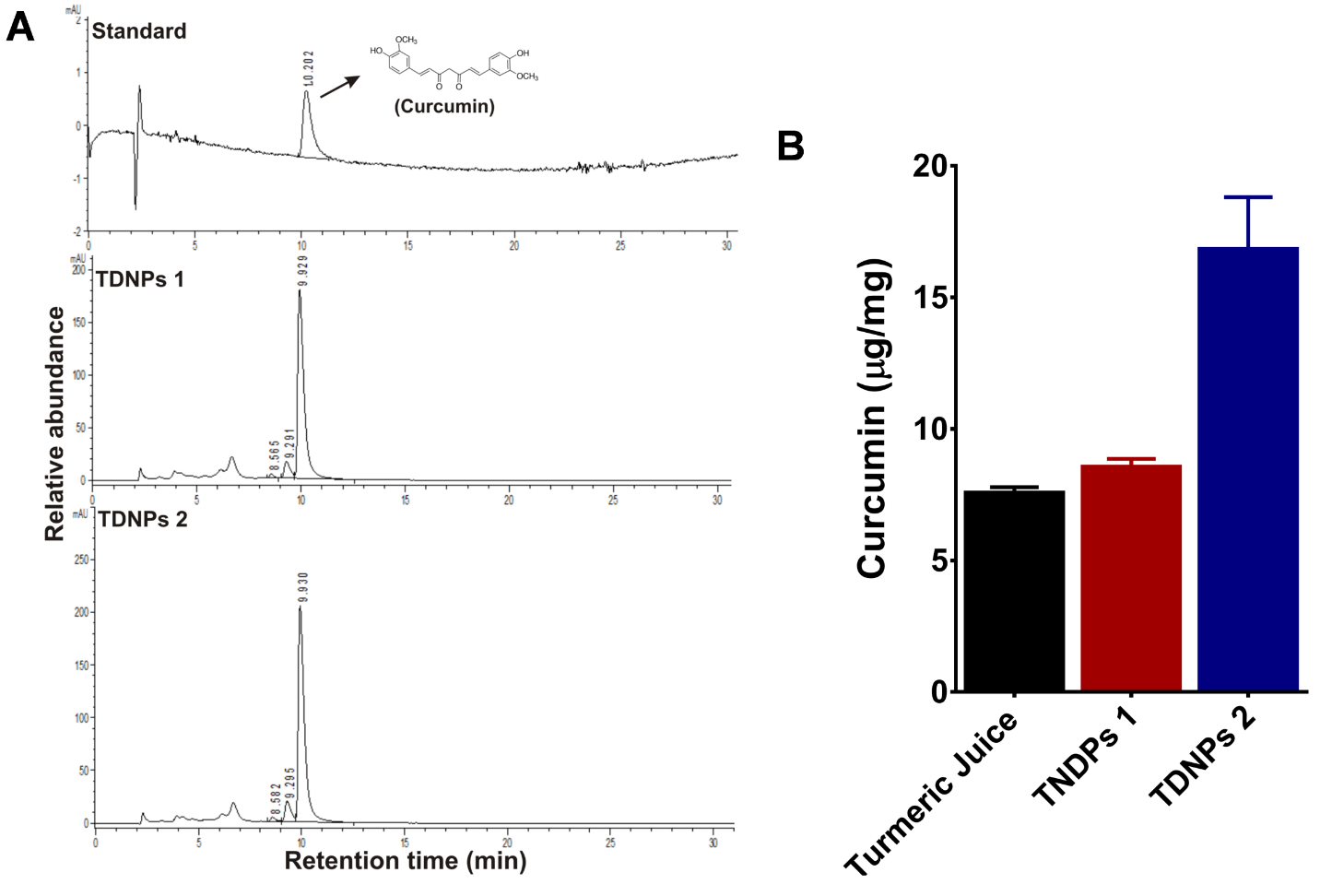


**Figure S2. Evaluation of the bioactive compound, curcumin, in turmeric juice and TNDPs by HPLC-UV.** (A) Analytical curve of standard curcumin, TNDPs 1 and TDNPs 2. (B) Curcumin content was normalized, n=3.


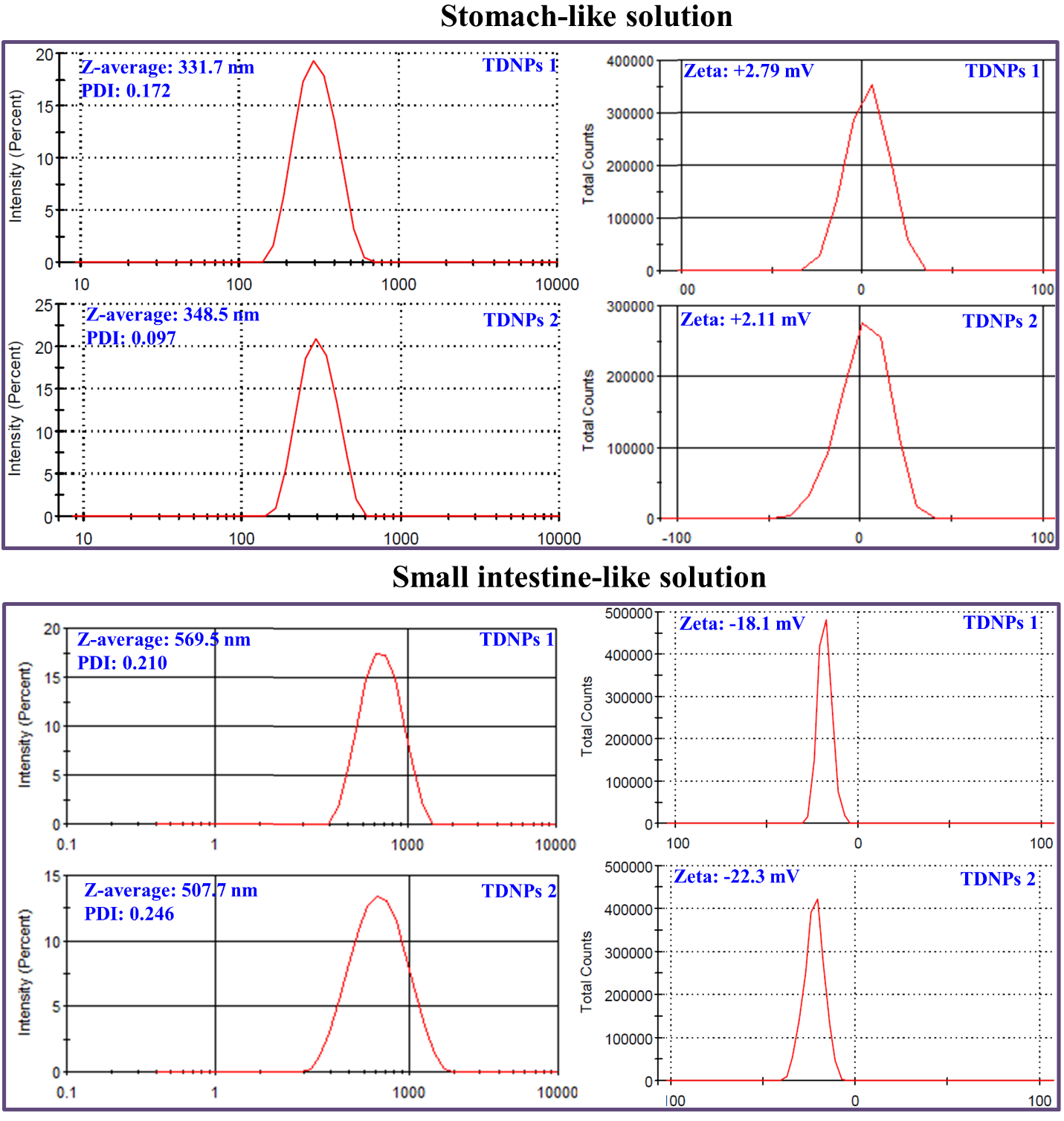


**Figure S3. Stability investigation of TDNPs.**


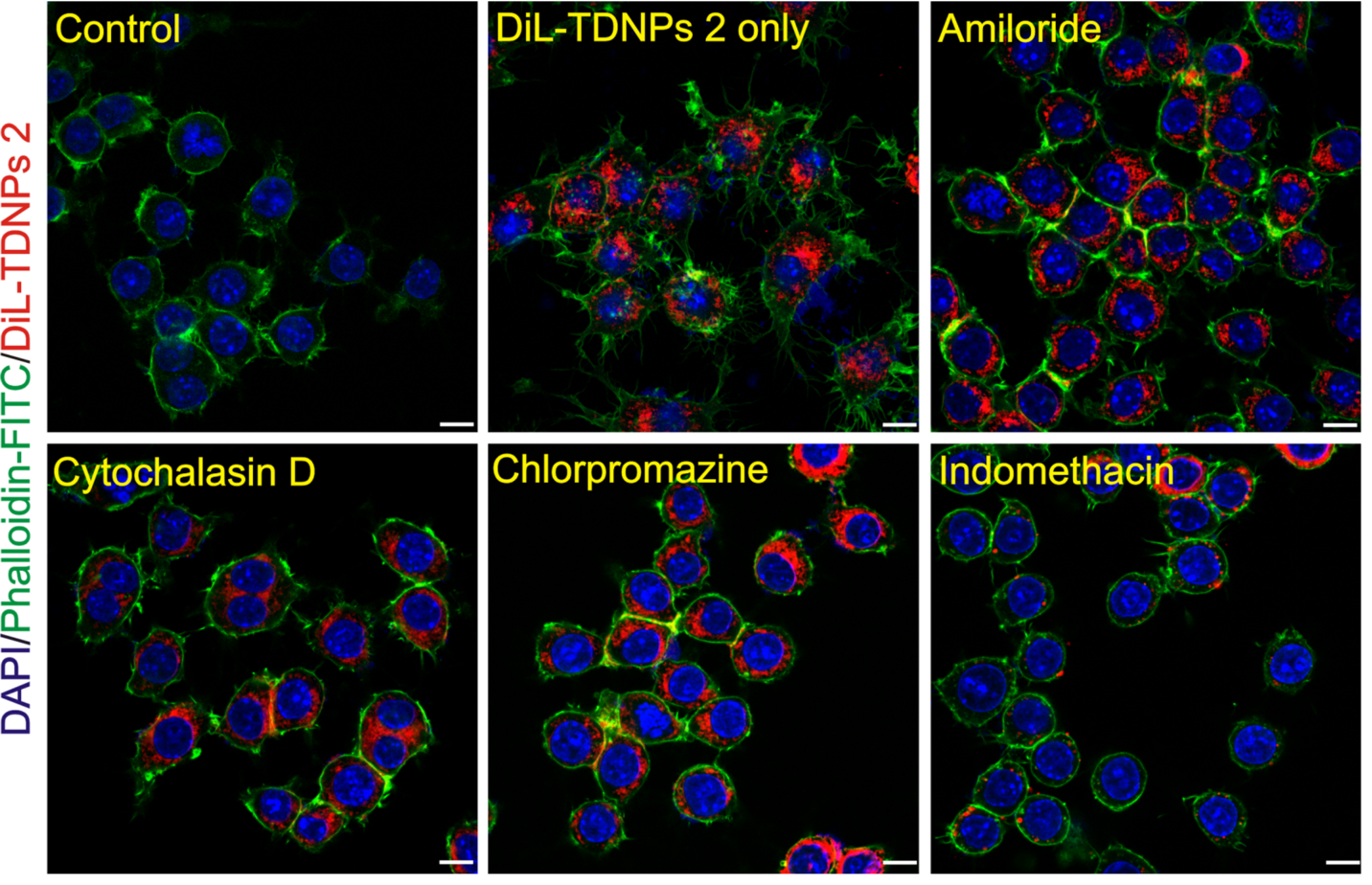


**Figure S4. Investigation the potential endocytosis pathway of TDNPs 2.** Special inhibitors were incubated with RAW 264.7 cells, then DiL-TDNPs 2 were added. Finally, cells were coverslip-mounted with DAPI for confocal imaging, scale bar: 20 μm.


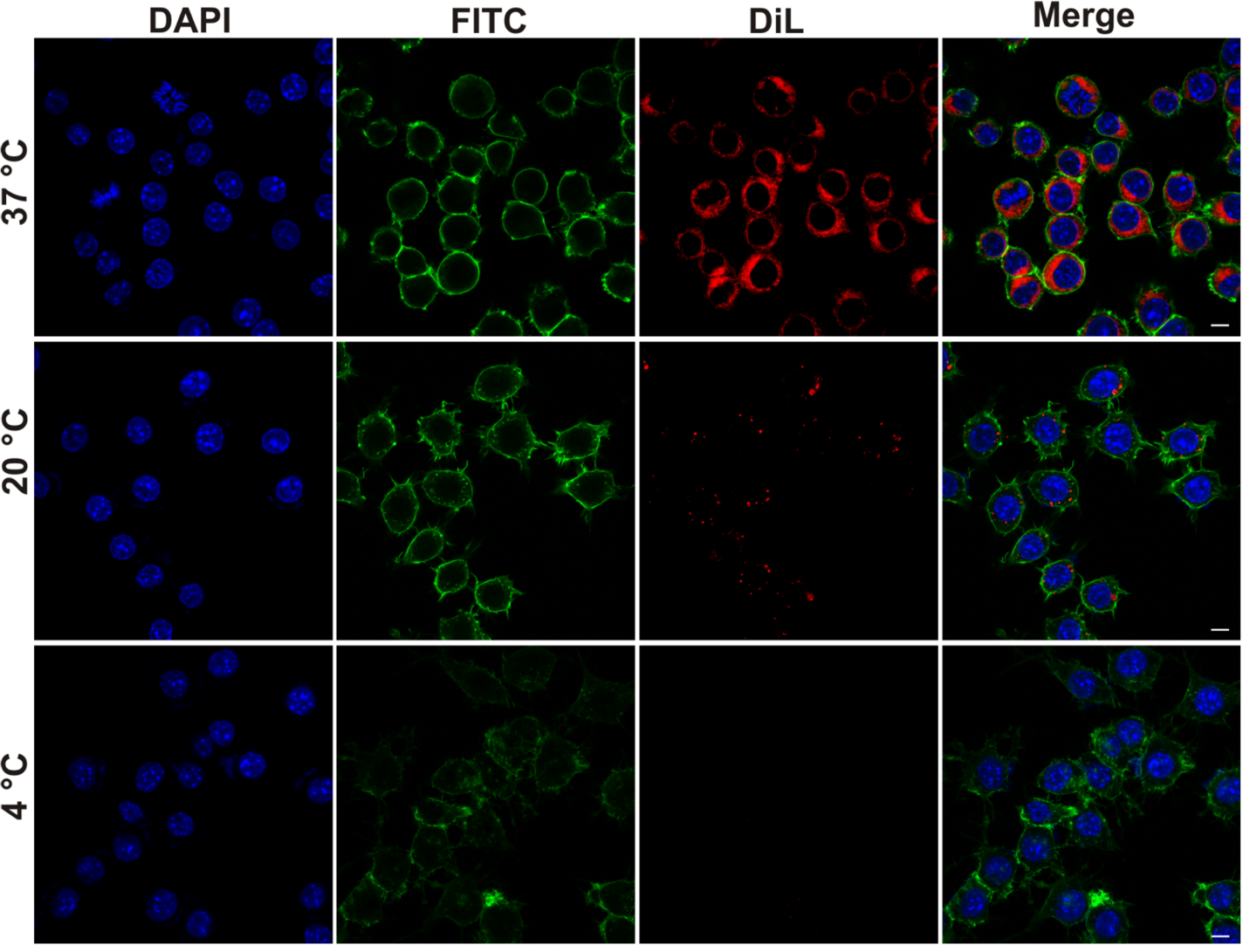


**Figure S5. Investigation the effect of temperature on TDNPs 2 internalization.** DiL-TDNPs 2 were incubated with RAW 264.7 cells under different temperatures, then cells were stained and imaged be confocal microscopy, scale bar: 20 μm.


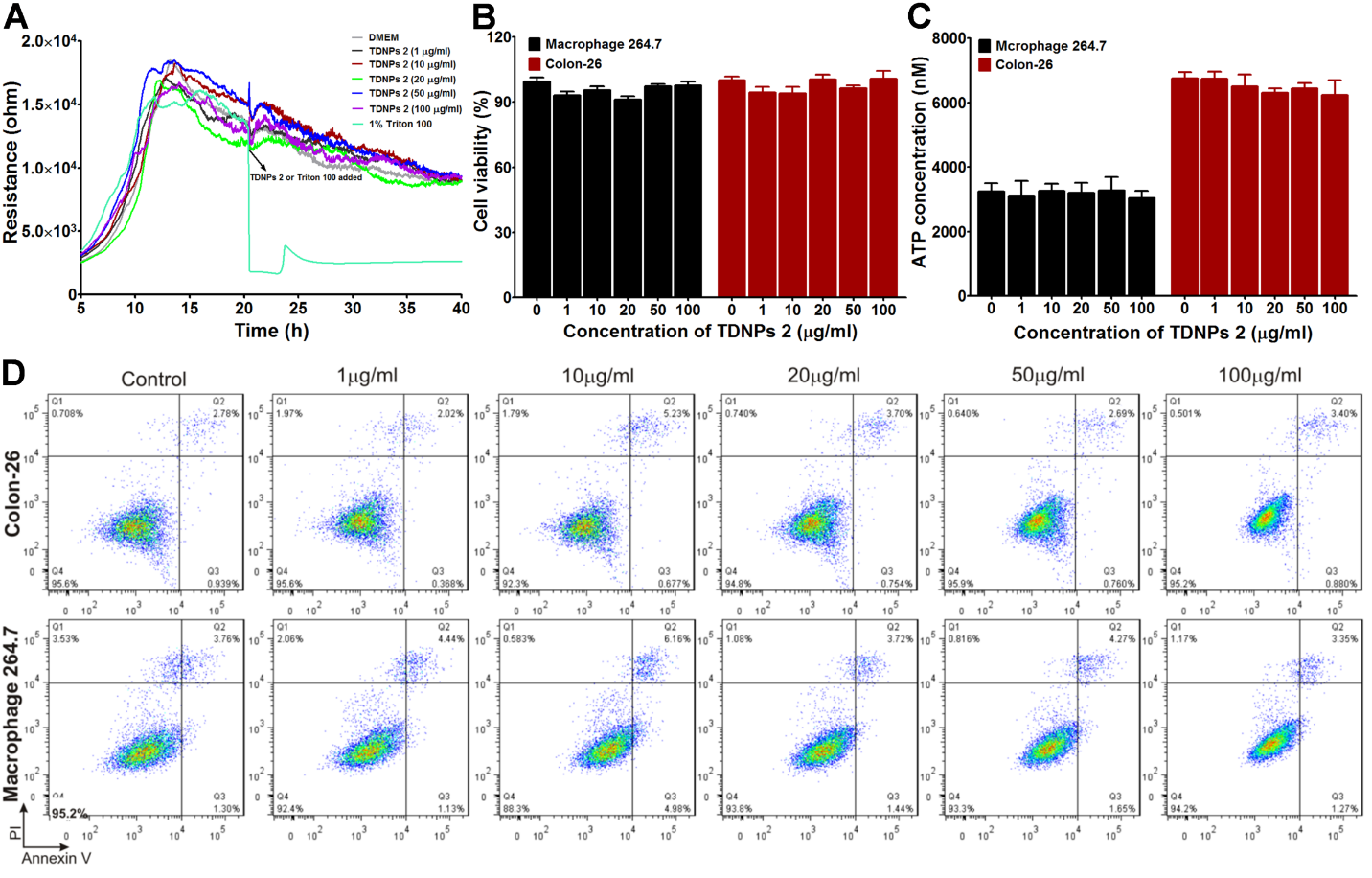


**Figure S6. Assess the biocompatibility of TDNPs 2 *in vitro*.** (A) Real-time *in vitro* cellular cytotoxicity of TNNPs 2 on Caco2 cells was monitored using electric cell-substrate impedance sensing (ECIS). (B) MTT assay was used to assess the potential toxicity of TDNPs 2 in macrophage 264.7 cells and colon-26 cells, (n=5). (C) Macrophage 264.7 and colon-26 cells proliferations were evaluated by ATPlite assays, (n=5). (D) Apoptosis of macrophage 264.7 and colon-26 cells was determined by Annexin V/PI stain assay, n=5.


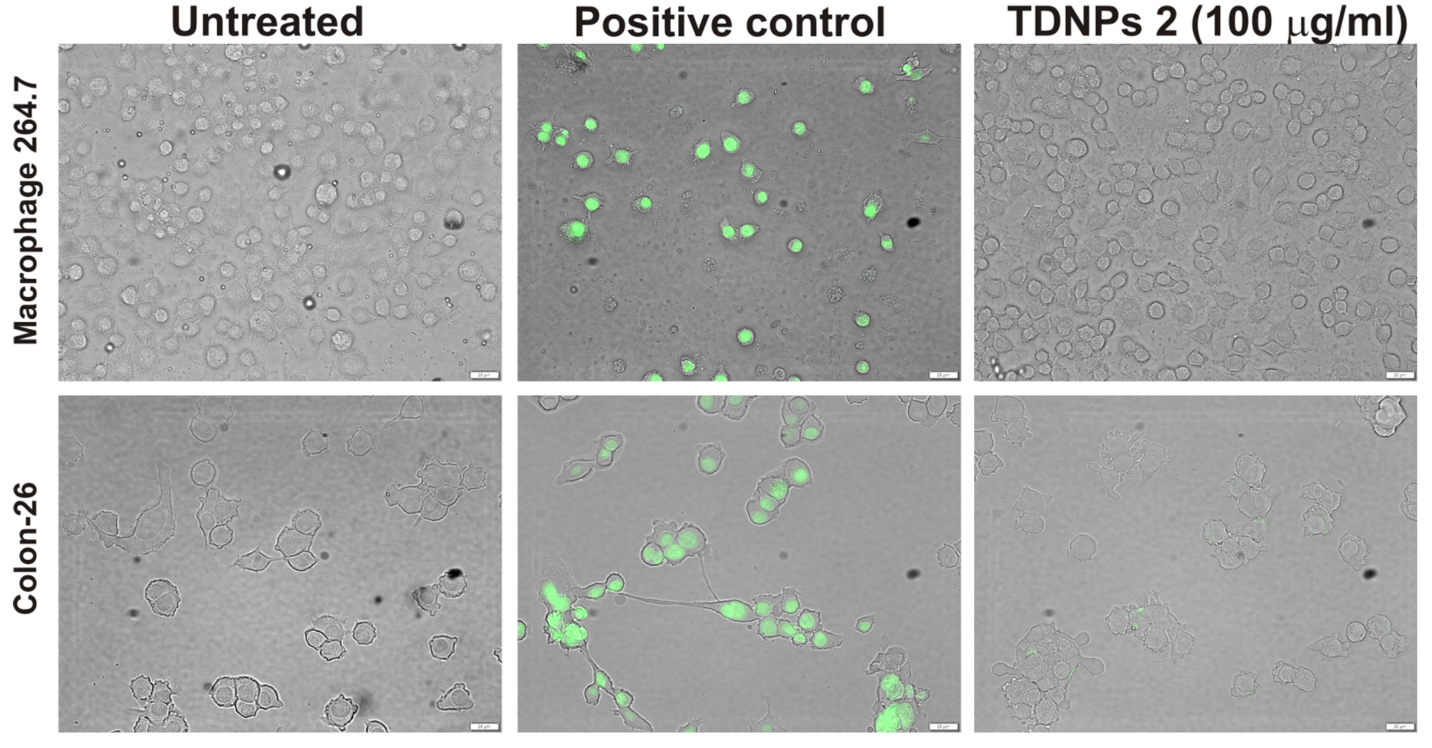


**Figure S7. Apoptosis of macrophage 264.7 and colon-26 cells was determined by the presence of activated caspase-3/7.** Apoptotic cells (green nuclei) were detected by staining for cleaved caspase-3/7, scale bar: 20 μm.

**Supplementary Tables**

**Table S1. Lipids found in Turmeric-derived nanoparticles.**

| **Name** | **Mass** | **Formula** | **Band 1 (n=5)^1^** | **Band 2 (n=5)^1^** |
| --- | --- | --- | --- | --- |
| **DGDG(34:6)** | 926.6 | C49H80O15 | 0.007 | 0.005 |
| **DGDG(34:5)** | 928.6 | C49H82O15 | 0.002 | 0.001 |
| **DGDG(34:4)** | 930.6 | C49H84O15 | 0.008 | 0.010 |
| **DGDG(34:3)** | 932.6 | C49H86O15 | 3.941 | 4.012 |
| **DGDG(34:2)** | 934.6 | C49H88O15 | 13.285 | 11.089 |
| **DGDG(34:1)** | 936.6 | C49H90O15 | 8.441 | 6.710 |
| **DGDG(36:6)** | 954.6 | C51H84O15 | 0.498 | 0.746 |
| **DGDG(36:5)** | 956.6 | C51H86O15 | 1.938 | 2.041 |
| **DGDG(36:4)** | 958.6 | C51H88O15 | 5.981 | 5.318 |
| **DGDG(36:3)** | 960.6 | C51H90O15 | 5.268 | 4.151 |
| **DGDG(36:2)** | 962.6 | C51H92O15 | 6.467 | 4.509 |
| **DGDG(36:1)** | 964.7 | C51H94O15 | 2.373 | 1.578 |
| **DGDG(38:6)** | 982.6 | C53H88O15 | 0.138 | 0.125 |
| **DGDG(38:5)** | 984.6 | C53H90O15 | 0.082 | 0.027 |
| **DGDG(38:4)** | 986.6 | C53H92O15 | 0.696 | 0.378 |
| **DGDG(38:3)** | 988.7 | C53H94O15 | 2.112 | 0.892 |
| **Total DGDG** |  |  | **51.237** | **41.592** |
| **MGDG(34:6)** | 764.5 | C43H70O10 | 0.000 | 0.001 |
| **MGDG(34:5)** | 766.5 | C43H72O10 | 0.001 | 0.002 |
| **MGDG(34:4)** | 768.5 | C43H74O10 | 0.008 | 0.015 |
| **MGDG(34:3)** | 770.5 | C43H76O10 | 1.369 | 1.076 |
| **MGDG(34:2)** | 772.6 | C43H78O10 | 1.712 | 0.997 |
| **MGDG(34:1)** | 774.6 | C43H80O10 | 1.462 | 0.866 |
| **MGDG(36:6)** | 792.5 | C45H74O10 | 0.810 | 1.860 |
| **MGDG(36:5)** | 794.5 | C45H76O10 | 1.557 | 2.699 |
| **MGDG(36:4)** | 796.6 | C45H78O10 | 2.701 | 2.671 |
| **MGDG(36:3)** | 798.6 | C45H80O10 | 1.475 | 1.157 |
| **MGDG(36:2)** | 800.6 | C45H82O10 | 0.833 | 0.543 |
| **MGDG(36:1)** | 802.6 | C45H84O10 | 0.421 | 0.247 |
| **MGDG(38:6)** | 820.6 | C47H78O10 | 0.000 | 0.000 |
| **MGDG(38:5)** | 822.6 | C47H80O10 | 0.000 | 0.000 |
| **MGDG(38:4)** | 824.6 | C47H82O10 | 0.078 | 0.037 |
| **MGDG(38:3)** | 826.6 | C47H84O10 | 0.215 | 0.153 |
| **Total MGDG** |  |  | **12.644** | **12.324** |
| **PG(32:1)** | 738.5 | C38H73O10P | 0.002 | 0.007 |
| **PG(32:0)** | 740.5 | C38H75O10P | 0.229 | 0.270 |
| **PG(34:4)** | 760.5 | C40H71O10P | 0.000 | 0.000 |
| **PG(34:3)** | 762.5 | C40H73O10P | 0.043 | 0.028 |
| **PG(34:2)** | 764.5 | C40H75O10P | 0.282 | 0.249 |
| **PG(34:1)** | 766.5 | C40H77O10P | 0.090 | 0.109 |
| **PG(34:0)** | 768.5 | C40H79O10P | 0.039 | 0.030 |
| **PG(36:6)** | 784.5 | C42H71O10P | 0.000 | 0.000 |
| **PG(36:5)** | 786.5 | C42H73O10P | 0.000 | 0.001 |
| **PG(36:4)** | 788.5 | C42H75O10P | 0.003 | 0.004 |
| **PG(36:3)** | 790.5 | C42H77O10P | 0.001 | 0.001 |
| **PG(36:2)** | 792.5 | C42H79O10P | 0.007 | 0.008 |
| **PG(36:1)** | 794.6 | C42H81O10P | 0.001 | 0.002 |
| **Total PG** |  |  | **0.696** | **0.709** |
| **LPG(16:1)** | 500.3 | C22H43O9P | 0.019 | 0.000 |
| **LPG(16:0)** | 502.3 | C22H45O9P | 0.064 | 0.093 |
| **LPG(18:3)** | 524.3 | C24H43O9P | 0.102 | 0.164 |
| **LPG(18:2)** | 526.3 | C24H45O9P | 0.068 | 0.029 |
| **LPG(18:1)** | 528.3 | C24H47O9P | 0.017 | 0.022 |
| **Total LysoPG** |  |  | **0.271** | **0.308** |
| **LPC(16:1)** | 494.3 | C24H48O7PN | 0.000 | 0.000 |
| **LPC(16:0)** | 496.3 | C24H50O7PN | 0.100 | 0.078 |
| **LPC(18:3)** | 518.3 | C26H48O7PN | 0.005 | 0.004 |
| **LPC(18:2)** | 520.3 | C26H50O7PN | 0.131 | 0.060 |
| **LPC(18:1)** | 522.3 | C26H52O7PN | 0.038 | 0.007 |
| **LPC(18:0)** | 524.4 | C26H54O7PN | 0.034 | 0.017 |
| **Total LysoPC** |  |  | **0.307** | **0.166** |
| **LPE(16:1)** | 452.3 | C21H42O7PN | 0.003 | 0.006 |
| **LPE(16:0)** | 454.3 | C21H44O7PN | 0.007 | 0.013 |
| **LPE(18:3)** | 476.3 | C23H42O7PN | 0.046 | 0.129 |
| **LPE(18:2)** | 478.3 | C23H44O7PN | 0.004 | 0.010 |
| **LPE(18:1)** | 480.3 | C23H46O7PN | 0.001 | 0.000 |
| **Total LysoPE** |  |  | **0.061** | **0.158** |
| **PC(32:0)** | 734.6 | C40H80O8PN | 0.065 | 0.074 |
| **PC(34:4)** | 754.5 | C42H76O8PN | 0.006 | 0.013 |
| **PC(34:3)** | 756.5 | C42H78O8PN | 0.746 | 1.011 |
| **PC(34:2)** | 758.6 | C42H80O8PN | 5.276 | 7.075 |
| **PC(34:1)** | 760.6 | C42H82O8PN | 0.492 | 0.646 |
| **PC(36:6)** | 778.5 | C44H76O8PN | 0.038 | 0.063 |
| **PC(36:5)** | 780.5 | C44H78O8PN | 0.502 | 0.776 |
| **PC(36:4)** | 782.6 | C44H80O8PN | 2.111 | 3.460 |
| **PC(36:3)** | 784.6 | C44H82O8PN | 0.540 | 0.898 |
| **PC(36:2)** | 786.6 | C44H84O8PN | 0.596 | 0.950 |
| **PC(36:1)** | 788.6 | C44H86O8PN | 0.086 | 0.140 |
| **PC(38:6)** | 806.6 | C46H80O8PN | 0.001 | 0.001 |
| **PC(38:5)** | 808.6 | C46H82O8PN | 0.008 | 0.004 |
| **PC(38:4)** | 810.6 | C46H84O8PN | 0.021 | 0.043 |
| **PC(38:3)** | 812.6 | C46H86O8PN | 0.062 | 0.111 |
| **PC(38:2)** | 814.6 | C46H88O8PN | 0.116 | 0.177 |
| **PC(40:5)** | 836.6 | C48H86O8PN | 0.000 | 0.000 |
| **PC(40:4)** | 838.6 | C48H88O8PN | 0.000 | 0.000 |
| **PC(40:3)** | 840.6 | C48H90O8PN | 0.002 | 0.003 |
| **PC(40:2)** | 842.7 | C48H92O8PN | 0.023 | 0.021 |
| **Total PC** |  |  | **10.692** | **15.467** |
| **PE(32:3)** | 686.5 | C37H68O8PN | 0.000 | 0.000 |
| **PE(32:2)** | 688.5 | C37H70O8PN | 0.000 | 0.001 |
| **PE(32:1)** | 690.5 | C37H72O8PN | 0.000 | 0.000 |
| **PE(32:0)** | 692.5 | C37H74O8PN | 0.000 | 0.001 |
| **PE(34:4)** | 712.5 | C39H70O8PN | 0.000 | 0.000 |
| **PE(34:3)** | 714.5 | C39H72O8PN | 0.018 | 0.049 |
| **PE(34:2)** | 716.5 | C39H74O8PN | 0.160 | 0.411 |
| **PE(34:1)** | 718.5 | C39H76O8PN | 0.004 | 0.011 |
| **PE(36:6)** | 736.5 | C41H70O8PN | 0.000 | 0.000 |
| **PE(36:5)** | 738.5 | C41H72O8PN | 0.006 | 0.016 |
| **PE(36:4)** | 740.5 | C41H74O8PN | 0.034 | 0.100 |
| **PE(36:3)** | 742.5 | C41H76O8PN | 0.003 | 0.012 |
| **PE(36:2)** | 744.5 | C41H78O8PN | 0.011 | 0.022 |
| **PE(36:1)** | 746.6 | C41H80O8PN | 0.000 | 0.000 |
| **PE(38:6)** | 764.5 | C43H74O8PN | 0.000 | 0.000 |
| **PE(38:5)** | 766.5 | C43H76O8PN | 0.000 | 0.000 |
| **PE(38:4)** | 768.5 | C43H78O8PN | 0.000 | 0.001 |
| **PE(38:3)** | 770.6 | C43H80O8PN | 0.001 | 0.005 |
| **PE(40:3)** | 798.6 | C45H84O8PN | 0.000 | 0.001 |
| **PE(40:2)** | 800.6 | C45H86O8PN | 0.001 | 0.004 |
| **PE(42:4)** | 824.6 | C47H86O8PN | 0.000 | 0.000 |
| **PE(42:3)** | 826.6 | C47H88O8PN | 0.001 | 0.003 |
| **PE(42:2)** | 828.6 | C47H90O8PN | 0.014 | 0.040 |
| **Total PE** |  |  | **0.253** | **0.677** |
| **PI(32:3)** | 822.5 | C41H73O13P | 0.013 | 0.011 |
| **PI(32:2)** | 824.5 | C41H75O13P | 0.041 | 0.042 |
| **PI(32:1)** | 826.5 | C41H77O13P | 0.040 | 0.045 |
| **PI(32:0)** | 828.5 | C41H79O13P | 0.452 | 0.374 |
| **PI(34:4)** | 848.5 | C43H75O13P | 0.004 | 0.002 |
| **PI(34:3)** | 850.5 | C43H77O13P | 1.720 | 1.457 |
| **PI(34:2)** | 852.5 | C43H79O13P | 7.128 | 5.330 |
| **PI(34:1)** | 854.5 | C43H81O13P | 0.781 | 0.582 |
| **PI(36:6)** | 872.5 | C45H75O13P | 0.011 | 0.007 |
| **PI(36:5)** | 874.5 | C45H77O13P | 0.090 | 0.065 |
| **PI(36:4)** | 876.5 | C45H79O13P | 0.372 | 0.272 |
| **PI(36:3)** | 878.5 | C45H81O13P | 0.117 | 0.103 |
| **PI(36:2)** | 880.6 | C45H83O13P | 0.283 | 0.212 |
| **PI(36:1)** | 882.6 | C45H85O13P | 0.049 | 0.043 |
| **Total PI** |  |  | **11.099** | **8.547** |
| **PS(34:4)** | 756.5 | C40H70O10PN | 0.000 | 0.001 |
| **PS(34:3)** | 758.5 | C40H72O10PN | 0.001 | 0.003 |
| **PS(34:2)** | 760.5 | C40H74O10PN | 0.007 | 0.039 |
| **PS(34:1)** | 762.5 | C40H76O10PN | 0.004 | 0.003 |
| **PS(36:6)** | 780.5 | C42H70O10PN | 0.000 | 0.000 |
| **PS(36:5)** | 782.5 | C42H72O10PN | 0.000 | 0.001 |
| **PS(36:4)** | 784.5 | C42H74O10PN | 0.001 | 0.003 |
| **PS(36:3)** | 786.5 | C42H76O10PN | 0.001 | 0.001 |
| **PS(36:2)** | 788.5 | C42H78O10PN | 0.001 | 0.006 |
| **PS(36:1)** | 790.6 | C42H80O10PN | 0.000 | 0.000 |
| **PS(38:6)** | 808.5 | C44H74O10PN | 0.000 | 0.000 |
| **PS(38:5)** | 810.5 | C44H76O10PN | 0.000 | 0.000 |
| **PS(38:4)** | 812.5 | C44H78O10PN | 0.000 | 0.000 |
| **PS(38:3)** | 814.6 | C44H80O10PN | 0.000 | 0.001 |
| **PS(38:2)** | 816.6 | C44H82O10PN | 0.000 | 0.001 |
| **PS(38:1)** | 818.6 | C44H84O10PN | 0.000 | 0.001 |
| **PS(40:4)** | 840.6 | C46H82O10PN | 0.000 | 0.000 |
| **PS(40:3)** | 842.6 | C46H84O10PN | 0.000 | 0.001 |
| **PS(40:2)** | 844.6 | C46H86O10PN | 0.002 | 0.022 |
| **PS(40:1)** | 846.6 | C46H88O10PN | 0.001 | 0.000 |
| **PS(42:4)** | 868.6 | C48H86O10PN | 0.000 | 0.000 |
| **PS(42:3)** | 870.6 | C48H88O10PN | 0.019 | 0.043 |
| **PS(42:2)** | 872.6 | C48H90O10PN | 0.016 | 0.201 |
| **PS(42:1)** | 874.6 | C48H92O10PN | 0.002 | 0.008 |
| **PS(44:3)** | 898.6 | C50H92O10PN | 0.006 | 0.000 |
| **PS(44:2)** | 900.7 | C50H94O10PN | 0.000 | 0.011 |
| **Total PS** |  |  | **0.062** | **0.343** |
| **PA(32:0)** | 666.5 | C35H69O8P | 0.121 | 0.180 |
| **PA(34:6)** | 682.4 | C37H61O8P | 0.000 | 0.000 |
| **PA(34:5)** | 684.4 | C37H63O8P | 0.001 | 0.000 |
| **PA(34:4)** | 686.4 | C37H65O8P | 0.008 | 0.005 |
| **PA(34:3)** | 688.5 | C37H67O8P | 0.886 | 1.260 |
| **PA(34:2)** | 690.5 | C37H69O8P | 6.802 | 9.844 |
| **PA(34:1)** | 692.5 | C37H71O8P | 0.655 | 0.913 |
| **PA(36:6)** | 710.4 | C39H65O8P | 0.033 | 0.061 |
| **PA(36:5)** | 712.5 | C39H67O8P | 0.474 | 0.813 |
| **PA(36:4)** | 714.5 | C39H69O8P | 2.454 | 4.593 |
| **PA(36:3)** | 716.5 | C39H71O8P | 0.663 | 1.073 |
| **PA(36:2)** | 718.5 | C39H73O8P | 0.581 | 0.968 |
| **Total PA** |  |  | **12.677** | **19.709** |
| **Total** |  |  | **100.000** | **100.000** |

^1^ The lipid composition of TDNPs was determined by using a triple quadrupole mass spectrometer. The data are as % of total signal for the molecular species determined after normalization of the signals in internal standards of the same lipid class. Lipid abbreviations: DGDG, digalactosyldiacylglycerol; MGDG, monogalactosyldiacylglycerol; PG, phosphatidylglycerol; LPG, lysophosphatidylglycerol; PC, phosphatidylcholine; LPC, lysophosphatidylcholine; PE, phosphatidylethanolamine; LPE, lysophosphatidylethanolamine; PI, phosphatidylinositol; PS, phosphatidylserine; PA, phosphatidic acid.

**Table S2: Proteomics analysis of TDNPs 2**

| **Accession Number** | **Protein description** |
| --- | --- |
| M0TZH4 | Aldose 1-epimerase |
| Q9TMQ7 | ATP synthase subunit beta |
| Q71N21 | Calmodulin |
| M0SHJ9 | Elongation factor Tu |
| M0TWS5 | Glyceraldehyde-3-phosphate dehydrogenase |
| M0RX74 | Glycylpeptide N-tetradecanoyltransferase |
| M0T172 | Kinesin-like protein |
| M0RXX8 | Lon protease homolog 2, peroxisomal |
| I1UDC5 | Maturase K |
| M0SWG6 | Potassium transporter |
| P82474 | Zingipain-2 |
| M0SA77 | Uncharacterized protein |
| M0TGC0 | Uncharacterized protein |
| M0SJG2 | Uncharacterized protein |
| M0RI40 | Uncharacterized protein |
| M0SGD4 | Uncharacterized protein |
| M0U3E2 | Uncharacterized protein |
| M0RJ46 | Uncharacterized protein |
| M0U305 | Uncharacterized protein |
| M0SDI3 | Uncharacterized protein |
| M0SNA3 | Uncharacterized protein |
| M0T1Z5 | Uncharacterized protein |
| M0RQI8 | Uncharacterized protein |
| M0SFW9 | Uncharacterized protein |
| M0RIC7 | Uncharacterized protein |
| M0U6P0 | Uncharacterized protein |
| M0SI17 | Uncharacterized protein |
| M0RZR7 | Uncharacterized protein |
| M0U2R3 | Uncharacterized protein |
| M0TQE9 | Uncharacterized protein |
| M0S453 | Uncharacterized protein |
| M0TTH7 | Uncharacterized protein |
| M0RIV7 | Uncharacterized protein |
| M0RY13 | Uncharacterized protein |
| M0TY03 | Uncharacterized protein |
| M0SQ97 | Uncharacterized protein |
| M0TVP4 | Uncharacterized protein |
| M0T6H9 | Uncharacterized protein |
| M0SMY9 | Uncharacterized protein |
| M0TN73 | Uncharacterized protein |
| M0UCD7 | Uncharacterized protein |
| M0RHX8 | Uncharacterized protein |
| M0TZJ0 | Uncharacterized protein |
| M0SGB7 | Uncharacterized protein |
| M0S7A6 | Uncharacterized protein |
| M0TX22 | Uncharacterized protein |

**Table S3. Primers used for Real-time PCR.**

| **Gene name** | **Forward primer (5’-3’)** | **Reverse primer(5’-3’)** |
| --- | --- | --- |
| **TNF-α** | AGGCTGCCCCGACTACGT | GACTTTCTCCTGGTATGAGATAGCAAA |
| **IL-6** | ACAAGTCGGAGGCTTAATTACACAT | TTGCCATTGCACAACTCTTTTC |
| **IL-1β** | TCGCTCAGGGTCACAAGAAA | CATCAGAGGCAAGGAGGA AAA C |
| **HO-1** | ACGCATATACCCGCTACCTG | CCAGAGTGTTCATTCGAGCA |
| **36b4** | TCCAGGCTTTGGGCATCA | CTTTATCAGCTGCACATCACTCAGA |
